# Tri‐WOB: A Bistability‐Driven Variable Stiffness Origami Structure for Soft Robotic Arms

**DOI:** 10.1002/advs.76898

**Published:** 2026-08-07

**Authors:** Lingchen Kong, Yaoyao Fiona Zhao

**Affiliations:** ^1^ Department of Mechanical Engineering McGill University Montreal Canada

**Keywords:** bistability, reconfiguration, soft robotic arms, variable stiffness, waterbomb origami

## Abstract

Soft robotic arms offer high compliance but suffer from limited load‐bearing capacity and energy‐intensive stiffness programming. Here, we present a bistability‐enabled origami structure, Tri‐WOB, composed of three Waterbomb‐origami‐based (WOB) units that achieve rapid, discrete, and energy‐free stiffness reconfiguration. When stacked into a modular robotic arm, Tri‐WOB enables large deformation while allowing stiffness modulation to define discrete end configurations, thereby improving positioning accuracy and repeatability. In the fully stiff configuration, the arm supports loads an order of magnitude greater than in the compliant state, while hybrid stiffness states preserve load‐bearing capacity during controlled directional bending. By leveraging structural bistability rather than powered stiffening mechanisms, this work proposes a novel design for energy‐efficient variable stiffness in origami robotics.

## Graphical abstract

Inherent bistability in Waterbomb origami enables the Tri‐WOB to switch between compliant and stiff states. By stacking multiple modules, the robotic arm maintains stiffness without continuous energy input while achieving large contraction, free morphing, and load‐bearing/transportation capability. This design provides a simple route toward versatile, strong, and reconfigurable soft robotic arms.

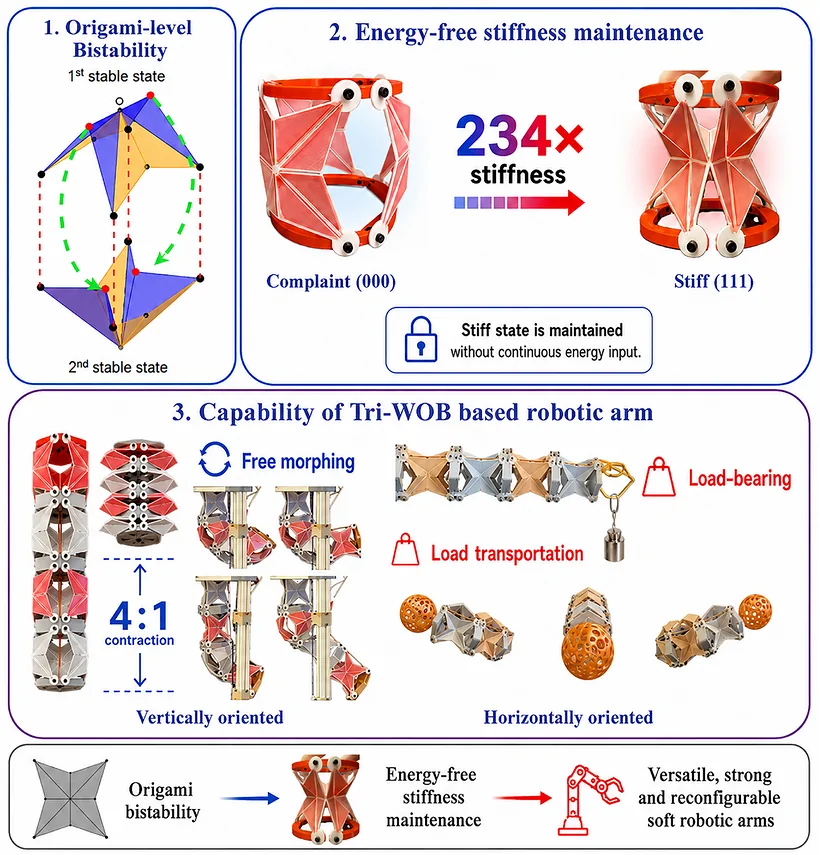

## 1 Introduction

Unlike traditional rigid‐bodied robots, soft robots constructed from compliant elastic materials can interact safely with their surroundings, enabling new modes of human–robot interaction and operation in unstructured environments [1, 2, 3]. A primary design objective for soft robots, especially soft robotic arms (SRAs), is to incorporate a large number of degrees of freedom (DoFs), allowing adaptive conformity around targets with various shapes and facilitating operation in challenging environments. Origami, the ancient art of paper folding, has emerged as a powerful design paradigm for SRAs due to its ability to transform flat two‐dimensional sheets into complex three‐dimensional structures through large, yet well‐defined deformations. Many origami patterns can be tessellated into tubular structures capable of axial extension, contraction, bending, and twisting with high deployment ratios [4, 5, 6, 7, 8, 9, 10]. Among these, Kresling origami is particularly notable for its intrinsic coupling between axial displacement and rotational motion, making it a popular choice for SRAs and linear actuators [11, 12, 13, 14, 15, 16].

Beyond flexibility, an equally critical capability is to rapidly switch between compliant and rigid states, as seen in biological soft tissues: elephant trunks and octopus tentacles can manipulate objects gently, yet stiffen quickly to generate large forces when needed. In contrast to continuum SRAs that typically distribute deformation throughout the entire body, origami‐based robots primarily localize deformation along predefined crease lines, of which the spatial arrangement governs the global deformation modes and mechanical response [17]. These characteristics raise a fundamental question for origami‐inspired SRAs: how can compliance for adaptability be compatible with high stiffness for effective load‐bearing?

To address this challenge, various stiffness‐enhancing strategies have been proposed for SRAs, which can broadly be categorized into two groups. The first category relies on integrating additional powered subsystems. One approach focuses on stimuli‐responsive materials, such as shape memory alloys (SMAs) [18, 19], shape memory polymers (SMPs) [20, 21, 22], and liquid crystal elastomers (LCEs) [23], which modulate stiffness via thermally induced phase transitions. Although attractive, these material‐based approaches are often constrained by low energy density and a limited achievable stiffness range, which can hinder practical use. Another powered approach employs mechanical mechanisms, such as jamming (granular, layer, or fiber) [24], where vacuum‐induced compression increases frictional interactions and rapidly enhances stiffness while retaining substantial deformability. However, jamming‐based structures typically offer limited extensibility, which restricts their applicability in scenarios requiring high elongation ratios [25]. Antagonistic stiffening offers another widely used mechanism by actively generating opposing internal forces, but, as with jamming‐based approaches, maintaining a stiff configuration requires continuous actuation, making the method inherently energy inefficient.

To address the energy inefficiency inherent to continuously actuated stiffness programming strategies, bistability provides a promising alternative. Bistable structures possess two stable equilibrium states, each corresponding to a distinct geometric configuration and mechanical property. The transition between two equilibrium states involves large, rapid deformations over short timescales [26]. Notably, once a bistable structure reaches either equilibrium, it can maintain its configuration without further energy input [27]. In origami systems, bistability arises primarily from creases, where selectively switching certain creases between mountain and valley states enables reconfiguration of the global geometry and, consequently, the mechanical response of the structure. One class of studies exploits the intrinsic bistability of classical origami patterns, such as Waterbomb [28], Miura [29], and Yoshimura origami [30], without altering their original crease layouts. In these designs, local transitions of crease states are sufficient to drive the structure between multiple globally stable configurations, yielding substantial stiffness modulation. The second class of approaches seeks to further expand the accessible design space by modifying the original crease layouts. By introducing additional creases or rearranging existing ones, these modified origami patterns enable a greater number of stable configurations and folding pathways, allowing more versatile post‐fabrication tuning of mechanical properties [31, 32, 33, 34, 35, 36].

Despite these advances, most bistable origami based stiffness modulation strategies primarily target axial stiffness control. In contrast, bending stiffness enhancement in SRAs has largely relied on the previously discussed powered approaches. Relatively little attention has been devoted to leveraging bistability as a mechanism for enhancing bending stiffness, particularly in a manner that eliminates the need for continuous actuation.

**FIGURE 1:**
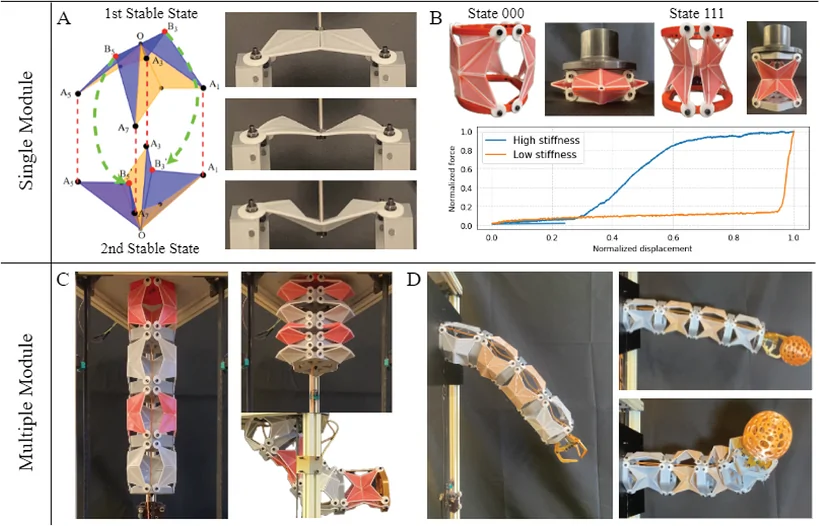
Waterbomb origami based Tri‐WOB structure and robotic arm demonstrations. (A) A single WOB unit, the fundamental building block of the Tri‐WOB, exhibiting two stable states and switching between them via downward compression of the central vertex. (B) Force–displacement responses of the Tri‐WOB in different stiffness states, demonstrating stiffness modulation. (C) Vertically oriented robotic arm assembled from modular Tri‐WOBs, illustrating a large compression ratio and wide deformation range. (D) Horizontally oriented robotic arm assembled from modular Tri‐WOBs, demonstrating enhanced bending stiffness under load.

In this work, we introduce a novel variable stiffness origami structure composed of three Waterbomb origami based (WOB) units, hence, referred to as Tri‐WOB. Owing to the inherent bistability of the Waterbomb geometry, each unit possesses two stable states and can switch between them via snap through by pressing the central vertex beyond the critical point (Figure 1A). Depending on the state of each unit, the assembled Tri‐WOB exhibits multiple discrete configurations. For example, State 000 denotes that all three units remain in the 1^st^ stable state, whereas State 111 indicates that all units have transitioned to the 2^nd^ stable state (Figure 1B). In State 000, the structure remains compliant and can deform freely under axial compression or bending. In contrast, switching to State 111 produces a substantial increase in both axial and radial stiffness with minimal change in height (Figure 1B and Movie S1). This bistable behavior enables rapid transitions between states while maintaining structural stability without continuous energy input.

As shown in Figure 1C, when multiple Tri‐WOBs are arranged axially, they form a modular SRA. Such a robotic arm demonstrates a wide deformation range, including significant axial contraction and “J‐type” bending in three directions driven by a three‐tendon actuation system. Moreover, without altering the actuation inputs, selectively switching the state of individual modules enables distinct target end postures. This discrete state control improves positional accuracy and repeatability by eliminating the intermediate uncertainty typically associated with continuous tendon‐driven deformation. In addition to enhancing configurational control, the proposed Tri‐WOB also enhances the bending stiffness of the robotic arm. When the arm is placed horizontally with all modules in State 000, it deflects easily under its own weight. In contrast, when all modules are switched to State 111, the arm exhibits minimal deflection even under an external load, demonstrating a great load‐bearing capacity improvement. Notably, the proposed design decouples the morphing capability from load‐bearing capacity. As shown in Figure 1D, the arm can sustain external loading while retaining large bending deformation, with tendon actuation primarily responsible for the deformation rather than load support.

Overall, the proposed Tri‐WOB establishes a new design that leverages bistability for stiffness modulation, providing two key advantages for SRAs. First, the modular Tri‐WOBs based robotic arm enables predictable and repeatable end configurations, thereby reducing actuator precision requirements. Second, it decouples morphing capability from load‐bearing capacity, allowing the arm to maintain structural strength while retaining large, controllable deformations.

## 2 Geometry Description and Bistability Analysis of WOB Unit

**FIGURE 2:**
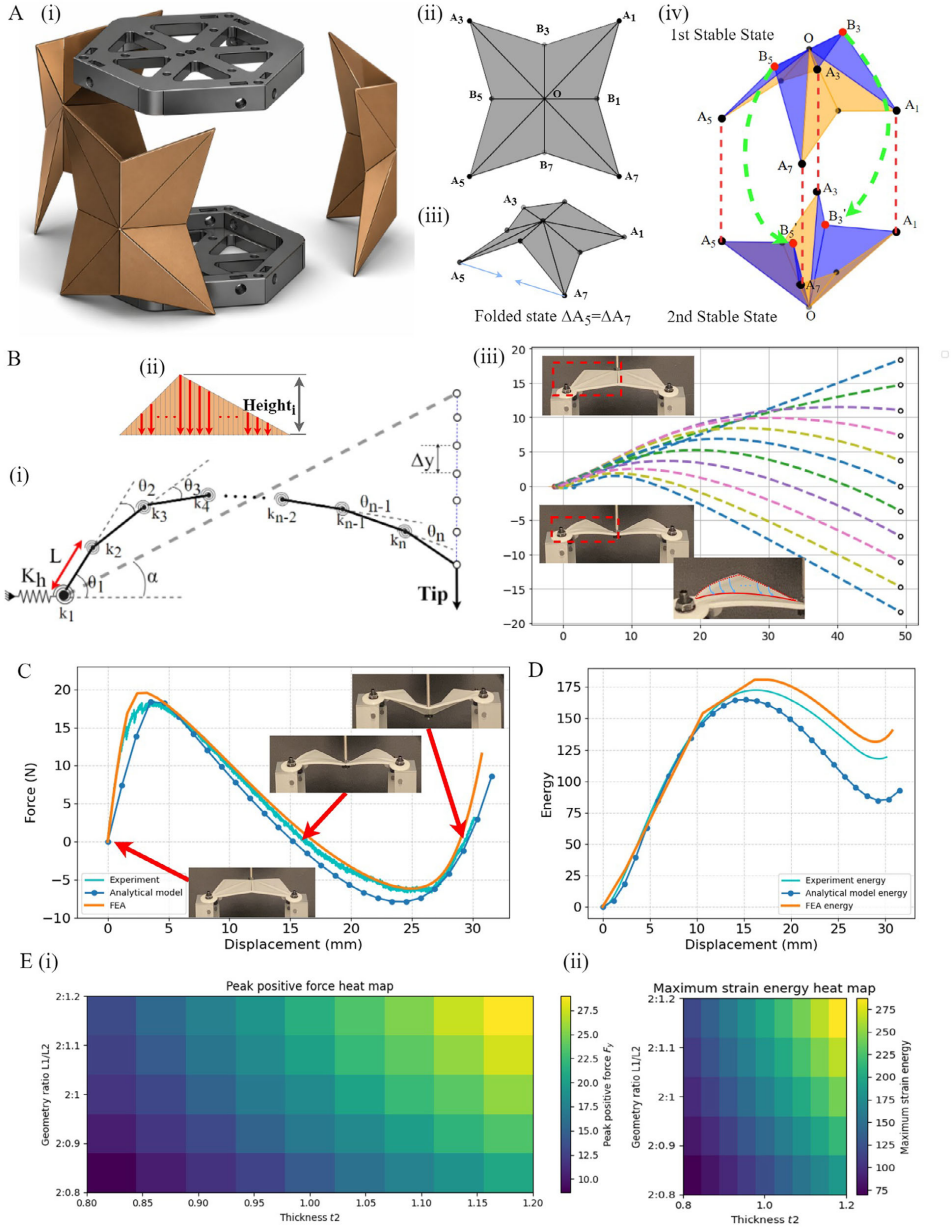
Modeling and analyses of the bistability of the WOB unit. (A) Geometric description of the Tri‐WOB and a single WOB unit: (i) construction of the Tri‐WOB; (ii) geometric description of a single WOB unit; (iii) initial folded state; (iv) diagram of two stable states. (B) Analytical model of the WOB unit: (i) multi‐segment cantilever beam representing a panel of the WOB unit; (ii) Panel height variation along the beam; (iii) simulated bistable process of the multi‐segment beam. (C) Force‐displacement curve of the bistable process, comparing experimental results, analytical predictions, and FEA. (D) Energy landscape of the WOB unit throughout the bistable process, with comparison among experiments, analytical modeling, and FEA. (E) Sensitivity analysis: effects of geometric ratio $L_{1}/L_{2}$ and PLA layer thickness $t_{2}$ on (i) the peak reaction force and (ii) the maximum stored strain energy during the bistable process.

The Tri‐WOB consists of two rigid frames and three WOB units, as shown in Figure 2A(i). The crease pattern of the WOB unit is illustrated in Figure 2A (ii). Each WOB unit has eight identical panels (denoted as $▵OA_{i}B_{i}$), where creases $OA_{i}$ and $OB_{i}$ connect adjacent panels. Creases are arranged such that adjacent lines are separated by $45^{\circ }$. The outer ends of $OA_{i}$ are referred to as external feet. After folding, $OA_{i}$ and $OB_{i}$ can deform to mountain folds or valley folds depending on the deformation modes. To be assembled to the Tri‐WOB, each WOB unit is folded from the flat state to gain the initial height by adjusting the distance between $A_{1}$ and $A_{3}$, and $A_{5}$ and $A_{7}$ ($D_{A_{1}A_{3}}=D_{A_{5}A_{7}}$), while the distance between $A_{1}$ and $A_{7}$, $A_{3}$ and $A_{5}$ remain unchanged (Figure 2A (iii)). After this adjustment, four external feet are fixed, where the current state of WOB unit is defined as the 1^st^ stable state. The bistability occurs when the vertex O undergoes a vertical downward displacement, as shown in Figure 2A (iv). After the snap through, all panels are below Plane $A_{1}A_{3}A_{5}A_{7}$ (Plane H), while 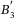 and 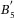 are still above Plane $OA_{1}A_{3}$ and Plane $OA_{5}A_{7}$. This configuration is referred to as the 2^nd^ stable state.

To capture the bistable process of the WOB unit from the 1^st^ stable state to the 2^nd^ stable state, an analytical model is developed. In conventional origami based bistability analyses, panels are typically treated as rigid bodies, while creases are modeled using rotational and extensional springs. The folded configuration is commonly taken as the initial state, implying zero initial strain energy. When an external force is applied, panels rotate about the creases and the creases extend, resulting in an increase of the system potential energy and driving the structure into an unstable state. As the applied force continues to increase, the stored potential energy surpasses the energy barrier, causing the structure to rapidly snap into the 2^nd^ stable state. A key assumption in these traditional models is that the external feet are free to move within a certain plane.

In the present study, however, the DoFs of the external feet are fully constrained, making the rigid panel assumption inappropriate. Under this constraint, the dominant source of stored potential energy arises from panel bending and buckling rather than crease rotation and extension. To simplify the problem, eight panels of the WOB unit are assumed to deform identically. Each panel is modeled as a multi‐segment cantilever beam, where individual segments remain rigid along the radial direction and are connected by rotational springs at their joints, as shown in Figure 2B(i). This formulation discretizes panel curvature into a series of straight segments (Figure 2B (ii)). Given the targeted position of the tip, the deformed shape of this multi‐segment beam can be obtained by finding a series of targeted angles $\theta_{i}$ around joints to minimize the total potential energy of the whole system. To capture the truth that the thinner section of the panel has a larger curvature, the rotational stiffness of the spring at each joint varies with the height of the section of the panel (Figure 2B (ii) and Figure S1).

Consequently, segments with smaller heights exhibit lower stiffness and undergo larger rotations under the same bending moment, enabling accurate representation of the natural curvature. Detailed settings of the analytical model can be found in Supplementary Text S1.

As the tip moves downward, the beam starts to deform, with the applied force at the tip increasing and exceeding the critical buckling force $F_{cr}$. The bistable process of the beam is illustrated in Figure 2B (iii), where larger deformation is observed near the foot sections, consistent with the proposed hypothesis. Beyond the critical point, the applied force decreases while the stored elastic energy continues to increase, indicating progression toward snap through. At the snap through point, the applied force reduces to zero while the stored energy reaches its maximum, corresponding to the configuration of maximum deformation. The system then rapidly transitions to the 2^nd^ stable state, during which the reaction force becomes negative, and the stored energy decreases.

To validate the analytical model, experiments are conducted on a WOB unit with the baseline geometric parameters. Each unit is modeled and 3D printed as a sandwich structure with two materials, two TPU layers on two sides and one PLA layer in the middle. The thicknesses of the TPU and PLA layers are $t_{1}=0.25$ mm and $t_{2}=1$ mm, respectively. For each panel $▵OA_{i}B_{i}$, $OA_{i}=L_{1}=52.5$ mm and $OB_{i}=L_{2}=26.25$ mm, giving a length ratio $L_{1}/L_{2}=2:1$. During testing, the WOB unit is fixed to a custom fixture and mounted on a compression testing machine. The central vertex of the unit is attached to the compression head to enable measurement of both positive and negative reaction forces. The force‐displacement curves during the bistable process are obtained by averaging three repeated experiments conducted at a loading rate of 5 mm/min, as shown in Figure 2C.

Finite element analysis (FEA) is also performed and the simulation results are presented in Figure 2C for comparison with the analytical predictions and experimental measurements. Both the analytical model and the FEA results show great agreement with the experimental data in terms of the initial stiffness, $F_{cr}$, the position of the snap through point and the end position of the bistable process. Detailed FEA settings are provided in Text S2.

To further validate the generality of the analytical model, additional experiments are conducted by varying both the geometric ratio and the panel thickness. Specifically, the length ratio $L_{1}/L_{2}$ varies from 2:0.8 to 2:1, to 2:1.2, while $t_{2}$ varies from 0.8 mm to 1 mm to 1.2 mm. In total, nine geometric configurations are fabricated and tested. The experimentally measured force–displacement curves are compared with the predictions of the analytical model, with the detailed comparisons provided in Figures S2–S4. The results demonstrate that the analytical model accurately predicts the bistable force– displacement behavior across different geometric configurations, confirming its generality while maintaining errors within an acceptable range.

Based on this validation, the analytical model is subsequently used to perform a broader sensitivity analysis. In this analysis, the geometric ratio $L_{1}/L_{2}$ is varied continuously from 2:0.8 to 2:1.2 with an interval of 0.1, while the thickness of the PLA layer $t_{2}$ varies from 0.8 to 1.2 mm with an interval of 0.05 mm. For each parameter combination, the peak reaction force and the maximum stored strain energy during the bistable process are calculated using the analytical model. The results are summarized as heatmaps in Figure 2E, revealing how geometric proportions and panel thickness jointly influence the bistable response of the WOB unit.

Cyclic testing is conducted to further evaluate the sensitivity of the bistability to fatigue. A total of 1000 loading–unloading cycles are performed on a WOB unit sample with the baseline geometric parameters, and the corresponding force–displacement curves are recorded, as shown in Figure S5A. A noticeable drift is observed during cycling. The positive peak force decreases from approximately 18 to 8 N, accompanied by a reduction in the initial stiffness, while the peak force position shifts slightly toward less displacement. As the number of cycles increases, the snap through point occurs at a smaller displacement, indicating that the second stable state is reached earlier. The negative peak force also decreases, from approximately 6 to 2.5 N, and its corresponding position shifts from 27.5 to 21 mm. In addition, the overall bistable process tends to complete earlier as cycling progresses.

Despite this degradation, the WOB unit still exhibits clear bistable behavior after 1000 cycles. A long‐time cyclic loading and large bending deformation around the foot sections result in irreversible plastic deformation of the panels, as shown in Figure S5C. Therefore, the reductions in initial stiffness and in both the positive and negative peak forces are mainly attributed to fatigue‐induced plastic deformation. It should be noted that the loading speed used in the cyclic test is 100 mm/min. In practical operation, the bistable process is manually triggered within approximately one second due to the snap through. Therefore, the deformation duration during the cyclic test is much longer than that experienced during normal operation. This extended loading duration may increase the accumulated plastic deformation and accelerate fatigue damage in the WOB unit. The detailed cyclic testing setup is provided in Text S3.

## 3 Performance of Tri‐WOB

The stiffness of the Tri‐WOB depends on the stable state of each WOB unit. When three units snap to the 2^nd^ stable state, the structure exhibits a significant increase in stiffness in both axial and radial directions. The global configuration is denoted by a three‐digit state descriptor: 000 indicates that all units remain in the 1^st^ stable state, whereas 111 indicates that all units are transitioned to the 2^nd^ stable state.

### 3.1 Global Variable Stiffness of Tri‐WOB

**FIGURE 3:**
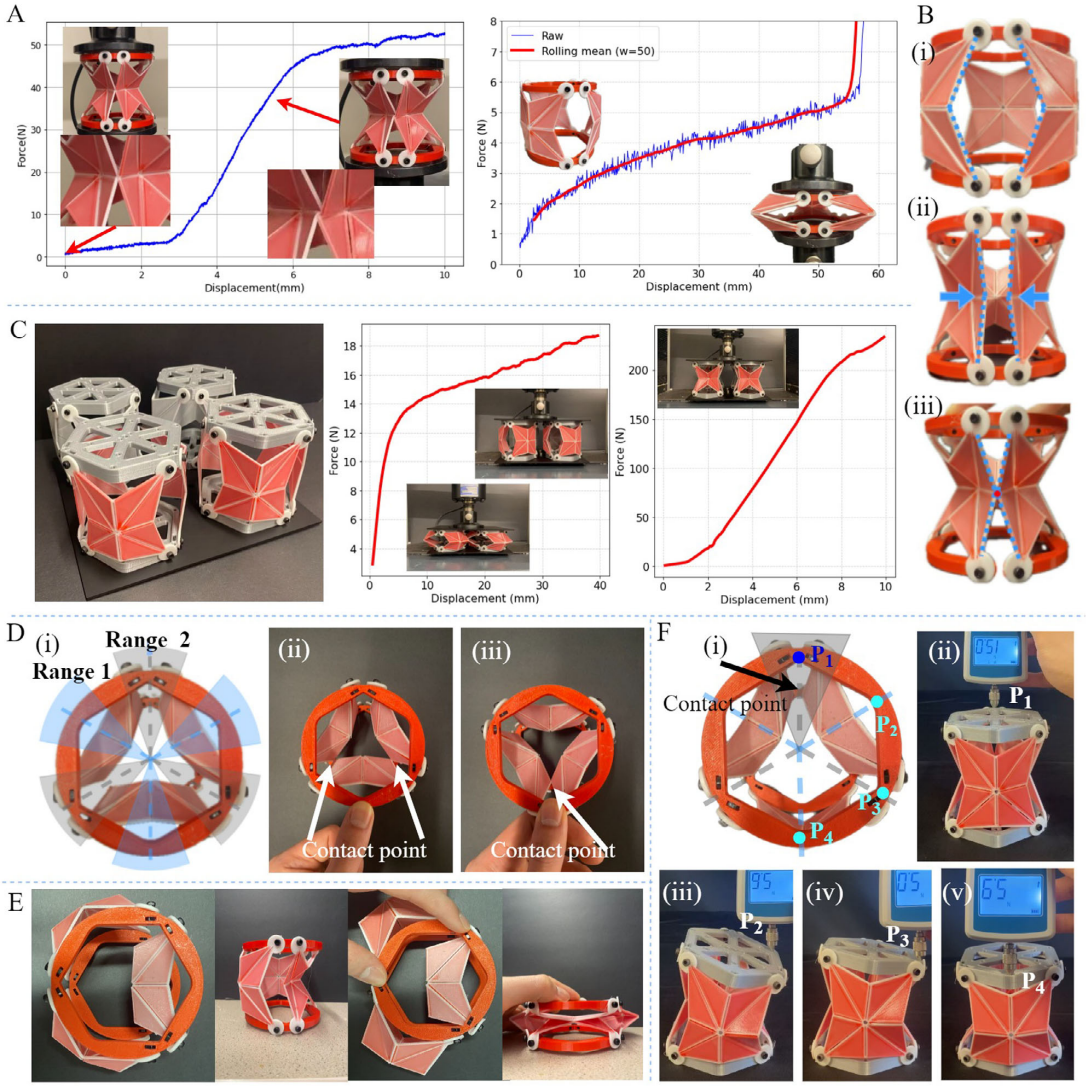
Mechanical performance of Tri‐WOB. (A) Force‐displacement curves of a single Tri‐WOB in State 000 and 111. (B) Stiffness enhancement mechanism: (i) initial edges resembling “<” and “>”, marked in blue dashed lines; (ii) geometric inversion during snap through; (iii) opposing vertex engagement under compression. (C) In‐plane 2×2 modularization and corresponding force–displacement responses in low and high stiffness states. (D) Effect of compression location for the Tri‐WOB in State 111: (i) definition of two loading regions; (ii) two vertex pairs engaged; (iii) one vertex pair engaged. (E) Mechanical response of the Tri‐WOB in State 100 under axial compression. (F) Non‐uniform stiffness distribution of the Tri‐WOB in State 101: (i) spatial definition of loading points $P_{1}$‐$P_{4}$; (ii‐v) mechanical responses of different loading points.

To quantify stiffness variation between State 000 and State 111, compression tests are performed on the Tri‐WOB. As shown in Figure 3A, in State 000, the Tri‐WOB exhibits low stiffness and can be easily compressed from $H_{1}=84$ mm to $H_{2}=20$ mm (compression ratio $H_{1}/H_{2}=4$). The maximum force is approximately 6 N, corresponding to an effective stiffness of 0.06 N/mm. However, after switching to State 111, the stiffness increases to 14.09 N/mm. With increasing displacement, the force enters a plateau region, where the maximum force is about 50 N. The stiffness ratio between the high and low stiffness states is therefore $K_{H}/K_{L}=14.09/0.06=234.83$.

The stiffness increase arises from the geometric interaction between adjacent WOB units. Before the snap through, the side vertices are positioned away from the center, where the corresponding edges form shapes resembling the symbol “<” and “>”, as shown in Figure 3B(i). Taking the left WOB unit as an example, when this unit snaps to the 2^nd^ stable state, the side vertex moves inward toward the center, where the shape of corresponding edges inverts from the “<” to “>” (Figure 3B (ii)). Under further compression, the vertex continues to move inward, and the dihedral angle associated with “>” decreases (Figure 3B (ii)). When neighboring units are also in the 2^nd^ stable state, the opposing side vertices engage. During compression, the left vertex tends to move right while the opposing vertex moves left, leading to mutual constraint (Figure 3B (iii)). As a result, as long as there is no relative movement between two side vertices, the Tri‐WOB can sustain high loads and exhibits high stiffness. Further compression will bend the panels corresponding to the opposing vertices, reducing the overall stiffness. However, in practice, fabrication imperfections prevent perfect alignment between opposing vertices. As shown in Movie S2, the vertices contact only briefly before relative slip occurs, which impacts the stiffness of the system and contributes the plateau region of Figure 3A.

Cyclic testing is also conducted on each of the three WOB units in the Tri‐WOB to evaluate the sensitivity of the assembled stiffness to fatigue. Three WOB units are separately subjected to 200, 500, and 1000 loading–unloading cycles and are then assembled into the Tri‐WOB for stiffness testing. As a baseline, the stiffness of the Tri‐WOB without cyclic loading is also measured. Therefore, four stiffness tests are performed for the Tri‐WOB assembled from WOB units after 0, 200, 500, and 1000 cycles, and the corresponding force–displacement curves are recorded, as shown in Figure S7. The stiffness is calculated from the slope of the linear region of each force–displacement curve. The slopes of the four curves remain close to each other, indicating that the overall stiffness of the Tri‐WOB is only slightly affected by cyclic fatigue. Specifically, the stiffness decreases from 15.26 N/mm at 0 cycles to 13.49 N/mm after 1000 cycles, corresponding to an 11.6% reduction and an 88.4% stiffness retention rate. However, the maximum force and the force level in the plateau region decrease as the number of cycles increases, which is closely related to the effectiveness of vertex engagement. Repeated cycling can increase the misalignment between opposing vertices, thereby weakening vertex contact. Nevertheless, the Tri‐WOB maintains a high stiffness level even after 1000 cycles, demonstrating good fatigue resistance and structural reliability.

After confirming the fatigue resistance of the Tri‐WOB, its modular scalability is further investigated by assembling multiple Tri‐WOBs in‐plane to enhance the load‐bearing capability of the overall system. To modularize Tri‐WOBs in‐plane, four Tri‐WOBs are equally distributed between the top and bottom plates and placed into the test machine. When all Tri‐WOBs are in State 000, the whole system can still be fully compressed easily. However, when all Tri‐WOBs are switched to State 111, the whole system demonstrates a higher stiffness than the single Tri‐WOB, where the whole system can sustain at least 200N force. The force‐displacement curves can be found in Figure 3C for both high and low stiffness situations.

### 3.2 Local Variable Stiffness of Tri‐WOB

The global stiffness increase arises from the interaction of three pairs of side vertices that come into contact under axial compression. When the loading condition shifts from uniform axial compression to localized point compression, the effective stiffness becomes dependent on the compression location. The compression location is divided into two regions, Range 1 (blue) and Range 2 (gray), as illustrated in Figure 3D(i). If the load is applied within Range 1, contact occurs between two pairs of side vertices, while the remaining pair remains separated (Figure 3D (ii)). In contrast, when the load is applied within Range 2, only one pair of side vertices comes into contact (Figure 3D (iii)). These observations indicate that the local stiffness enhancement depends on the number of engaged vertex pairs during compression.

Furthermore, the stiffness distribution is influenced by the stable state of the individual WOB units. When only one unit is switched to the 2^nd^ stable state (State 100), axial compression does not produce side‐vertex contact, and the stiffness remains unchanged and low (Figure 3E and Movie S3). However, when two units are switched (State 101, as shown in Figure 3F(i)), the stiffness becomes spatially non‐uniform (Movie S3). Specifically, stiffness increases in regions near the engaged side vertices of the switched units, where loads of up to 15N can be sustained (Figure 3F (ii)), whereas regions farther from the contact zone remain comparatively compliant, supporting only 5−6N (Figure 3F (iii–v)). Such direction‐dependent behavior paves the way for SRAs that decouple morphing capability from load‐bearing capacity, as discussed in the next section.

## 4 Tri‐WOB Based Robotic Arm

**FIGURE 4:**
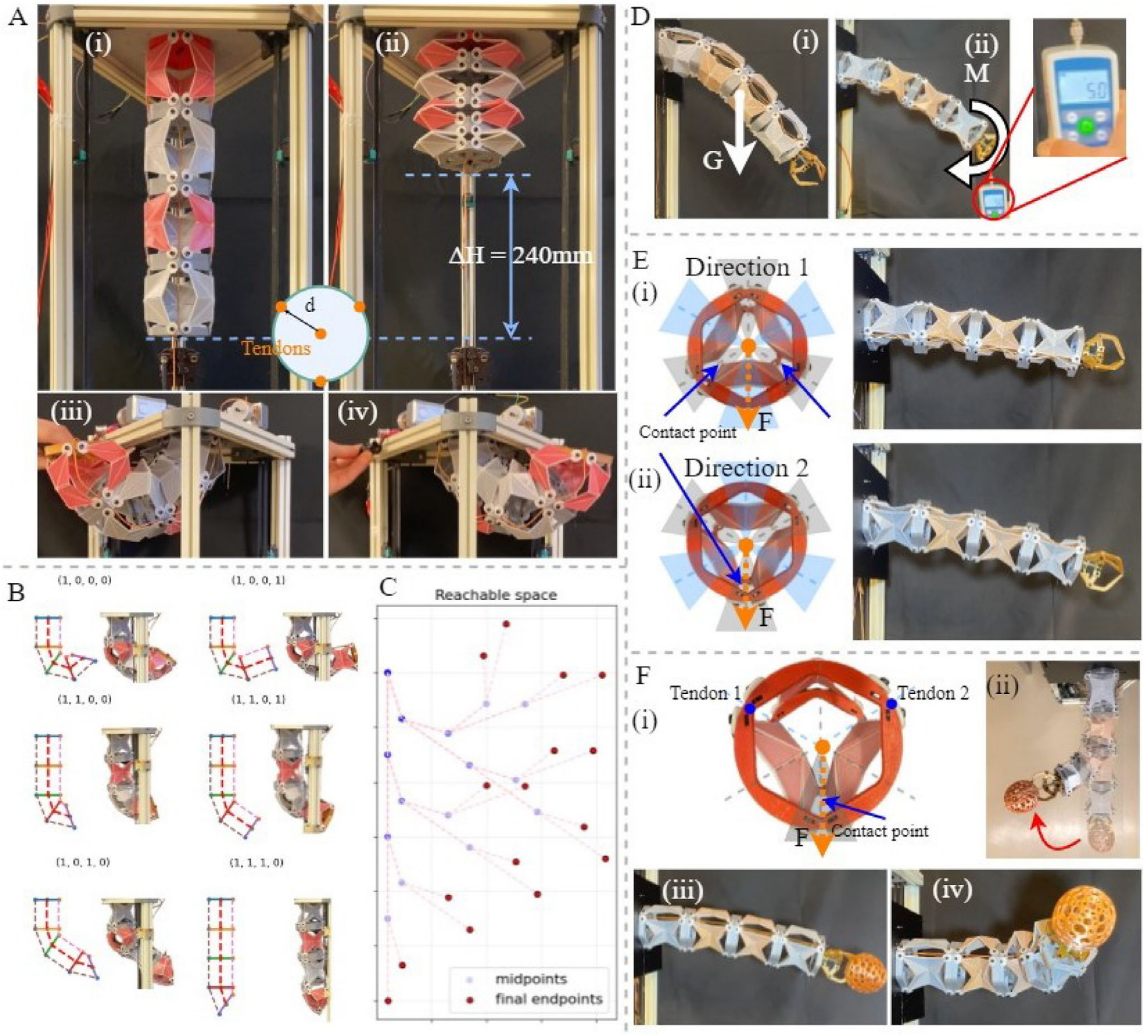
Performance of Tri‐WOB–based robotic arm. (A) Free morphing of four stacked Tri‐WOBs: (i) fully extended; (ii) fully contracted; (iii–iv) directional bending. (B) End postures of six representative configurations (out of 16 total configurations) under full tendon actuation. (C) Predicted reachable workspace of the robotic arm. (D) Global stiffness under horizontal loading: (i) compliant 0000 configuration exhibiting significant self‐weight deflection; (ii) stiff 1111 configuration supporting external load with reduced curvature. (E) Effect of module orientation: (i) Direction 1 arrangement with two vertex pairs engaged under downward loading; (ii) Direction 2 arrangement with one vertex pair engaged. (F) Spatially non‐uniform stiffness (State 101) enabling partial decoupling of deformation and load‐bearing: (i) bending schematic showing bottom vertex engagement and compliant upper pairs; (ii–iv) deformed arm supporting an external load.

As shown in Figure 4A, the robotic arm consists of four Tri‐WOBs stacked along the Z‐axis. The base of the arm is connected to a control module that consists of four motor‐driven actuation units. Three motors are positioned circumferentially at $120^{\circ }$ intervals to enable directional bending, while the fourth motor is connected to the central tendon to control axial contraction. Four tendons extend from the base to the distal end of the arm and serve as transmission elements for actuation. By coordinating tendons, the arm can achieve controlled bending and contraction. The following subsections evaluate the deformation ability of the proposed robotic arm and its load‐bearing performance under different stiffness configurations and loading conditions.

### 4.1 Free Morphing Capability and Posture Control

The free morphing capability of the Tri‐WOB–based robotic arm is first demonstrated with all modules in the compliant State 000, in which the arm exhibits continuous and highly compliant deformation. Activation of the motor connected to the central tendon initiates axial contraction by retracting the tendon. Over an actuation period of 13 s, the arm shortens from its maximum length of 320 mm (Figure 4A(i)) to a minimum length of 80 mm (Figure 4A (ii)), corresponding to a contraction ratio of 4:1 (Movie S4). Directional bending is achieved by activating the circumferentially positioned motors, which apply off‐axis tendon forces and induce asymmetric deformation, leading to a J‐type bending configuration. When the tendons are fully retracted, the distal end of the robotic arm can reach the same height level as the base, forming an arc of approximately $180^{\circ }$, as shown in Figure 4A (iii–iv) and Movie S4.

When the high‐stiffness State 111 is incorporated, the robotic arm can adopt 16 ($=2^{4}$) different configurations. For clarity, these configurations are labeled by (ijkl), where i,j,k,l∈{0,1} represent the stiffness state of each Tri‐WOB from base to tip (0: compliant State 000; 1: stiff State 111). Thus, configuration 0000 corresponds to a fully compliant arm capable of free morphing, whereas 1111 represents a fully stiff, immobile state. In every configuration, the end posture corresponds to the fully actuated condition in which the tendons are completely retracted. To determine these end postures, a kinematic simulation workflow is developed. Since the arm consists of serially stacked modules along the Z‐axis, only a single Tri‐WOB is modeled in Adams. In this model, the compliant State 000 module is represented as a 3‐RSR parallel mechanism and simulated from the straight configuration to its fully bent state, while the stiff State 111 module is simplified as a rigid column with a constant height of 84 mm. The simulation provides the deformation trajectory and final endpoint position of the compliant module (details in Text S4). The Adams output is then imported into a post‐processing script, where four modules are virtually stacked end‐to‐end to reconstruct the full arm. For each configuration, modules assigned to State 111 remain straight, whereas modules in State 000 adopt the simulated bent end pose. By sequentially transforming and rotating the module endpoints, the script computes the final tip position and visualizes the resulting arm configurations and reachable workspace, as illustrated in Figure 4C. To validate the predicted configurations, all 16 cases are experimentally tested. Six representative configurations are presented here for direct comparison, as shown in Figure 4B, while the complete set is provided in Figure S9. Minor discrepancies observed in the final four configurations arise primarily because gravitational effects are not included in the Adams simulations. Repeatability analysis is conducted to evaluate the end‐point position error of the robotic arm for seven representative configurations. Each configuration is activated 10 times, and the end‐point position is recorded when the robotic arm reaches its fully activated state. A marker is attached to the distal end of the robotic arm, and high‐resolution images are taken at the fully activated state. An image‐processing script is then used to determine the relative position of the marker with respect to the base point. The variation among the 10 repeated tests is subsequently calculated to assess the repeatability of the end‐point position. The test results are provided in Figure S10A–G. For all configurations, the maximum end‐point position error over 10 repeated tests is less than 5%, demonstrating great position‐holding capability.

Moreover, the grasping and load transportation capabilities are demonstrated in Movie S5 using configuration 1000. An electromagnetic plate is attached to the distal end of the robotic arm. The robotic arm is first activated to bend toward the left until it reaches the desired position, where the electromagnetic plate is activated to attract the object weighing 250 g. The robotic arm then returns to its initial position and bends toward the right. When it reaches the designed height, the electromagnetic plate is deactivated to release the object into the box. The trajectory‐holding capability under load is also analyzed. Videos are recorded under both loaded and unloaded conditions and processed using a video‐processing script. The end‐point position of the robotic arm is tracked during the tests, and the trajectories are extracted and shown in Figure S11A,B. The comparison shows that applying a 250 g load results in a 78.17 mm trajectory shift under the fully bent condition. Additionally, for each condition, the forward and return trajectories differ by up to 32 mm.

The Tri‐WOB enables the robotic arm with direct control of the end posture of the robotic arm through reconfiguration rather than continuous actuation. Although this strategy discretizes the set of achievable shapes, it significantly improves positional accuracy and repeatability by eliminating uncertainty associated with intermediate deformation states. By decoupling tendon actuation from final posture determination, the system simplifies control and avoids the complexity and unpredictability inherent in continuum robotic arms. In effect, the robotic arm trades unrestricted deformation freedom for enhanced controllability and precision, providing a practical balance between morphing capability and reliable positioning.

### 4.2 Bending Stiffness and Load‐Bearing Performance

When the robotic arm is oriented horizontally, it can be modeled as a cantilever beam. This section therefore focuses on bending stiffness and load‐bearing capacity. When all Tri‐WOBs are in State 000 (i.e. the robotic arm is in 0000 configuration), the arm exhibits low bending stiffness and undergoes noticeable deflection under its own weight, with the largest curvature occurring near the base (Figure 4D(i)). In this configuration, the load‐bearing capacity is limited. However, switching to high stiffness state enables a great stiffness increase to the robotic arm and can support a 5N load (Figure 4D (ii) and Movie S6).

Under gravity or an externally applied downward load, each horizontally oriented Tri‐WOB experiences a clockwise bending moment. Since the bending moment increases with distance from the load application point, the largest moment occurs at the base module. Consequently, the overall load‐bearing capacity of the robotic arm is limited by the moment resistance of this base module.

Similar to the localized point compression behavior described earlier, the effective stiffness depends on the location and extent of vertex contact. When all modules are oriented in Direction 1 (Figure 4E(i)), the downward force causes two pairs of side vertices to engage while the remaining pair remains separated. In contrast, when modules are oriented in Direction 2, only one vertex pair engages, as illustrated in Figure 4E (ii). Since vertex contact constrains relative motion, a greater number of engaged pairs results in higher bending stiffness for each module. As a result, the arm exhibits a smaller curvature in Direction 1 than in Direction 2 under its own weight. In both cases, the slight curvature observed under unloaded conditions originates from the initial gap between opposing side vertices when corresponding WOB units are in the 2^nd^ stable state (Figure 3B (ii)), which allows slight bending before vertex engagement occurs. Although the Direction 2 arrangement theoretically yields less bending stiffness, it offers the advantage of decoupling deformation capability from load‐bearing performance. As discussed in the previous section, this can be achieved by switching only the bottom two WOB units to the 2^nd^ stable state while keeping the top unit in the 1^st^ stable state (i.e., Tri‐WOB is in State 101, as shown in Figure 4F(i)), producing a spatially non‐uniform stiffness distribution. For the horizontally oriented arm, the bending stiffness remains high when applying a downward load and a clockwise bending moment, as this engages the bottom pair of opposing side vertices. In contrast, regions farther from the contact zone remain comparatively compliant. Consequently, considering the location of tendons, retracting one of the upper tendons allows the arm to bend toward the corresponding direction while the lower vertex contacts remain engaged. As shown in Figure 4F (ii–iv), when all Tri‐WOBs of the robotic arm are in State 101, the arm can support an object while still achieving controlled bending through selective tendon actuation (Movie S7). Importantly, whether the arm is in a straight or bent configuration, the load‐bearing capacity arises from the structural engagement of Tri‐WOBs, while the tendons serve solely as actuation elements.

However, such directional deformation inevitably compromises the load‐bearing capacity to some extent.

For a Tri‐WOB in State 101, bending along the Tendon 1 direction allows the left side vertex to move rightward, which in turn pushes the right side vertex further to the right without significant resistance. Consequently, the angle of the “<” shape formed by the right edges increases (Figure S12(i),(ii)). With progressive bending along the Tendon 1 direction, this angle continues to open, driving the shape “<” to the critical point resembling “|” (Figure S12(iii)), and eventually inverting into the shape “>” (Figure S12(iv)). Under compression along Tendon 3, corresponding to the downward load applied to the horizontal arm, the originally opposing side vertices become aligned in the same direction (forming “> >,” as shown in Figure S12(v)). This geometric change eliminates the engagement between the two opposing side vertices and the associated stiffness support. As a result, retraction of Tendon 1 (or Tendon 2) alters the shape “<” of the right (left) edges across all Tri‐WOBs, thereby reducing the bending stiffness and overall load‐bearing capacity. During the experiments, the load‐bearing capacity of the deformed robotic arm is found to be approximately 50 g. When a larger weight is applied at the distal tip, the engagement between the opposing side vertices of the base Tri‐WOB begins to fail, leading to the collapse of the robotic arm.

Repeatability analysis is also conducted for the horizontally oriented robotic arm without load using the same tracking protocol and image‐processing method described in Section 4.1. The repeated‐test results are shown in Figure S10H, where the maximum error is approximately 2.5%. The grasping and load transportation capabilities are demonstrated in movie S8. The same electromagnetic plate is used to attract and release an object weighing 50 g. The trajectory‐holding capability under load is also analyzed. Videos are recorded under both loaded and unloaded conditions and processed using the video‐processing script. The end‐point position of the robotic arm is tracked during the tests, and the trajectories are extracted and shown in Figure S11C,D. The comparison shows that applying a 50 g load results in a 20.4 mm trajectory shift under the bent condition. Additionally, unlike the vertically oriented case, the forward and return trajectories are close to each other under both loaded and unloaded conditions.

The performance of various robotic arms and related stiffness‐enhancement methods reported in the literature is summarized in Table S1, with a focus on the stiffness‐enhancement ratio, loading capacity, response time, energy required to maintain the stiff state, deformation range, and repeatability. The stiffness‐enhancement ratio and loading capacity are compared separately under lateral and axial loading conditions. The deformation range is evaluated based on the bending angle and the extension‐to‐compression ratio. Among the reported robotic arms, most rely on stiffness‐enhancement mechanisms that require continuous energy input, except for SMA‐ and LMPA‐based approaches, but with limited response time and deformation range. In contrast, the robotic arm proposed in this study takes advantage of the bistability of origami to achieve a promising stiffness‐enhancement ratio without continuous energy input.

Overall, the proposed Tri‐WOB enables the robotic arm to achieve a large deformation range while allowing discrete stiffness‐based reconfiguration for end posture control, thereby improving positioning accuracy and repeatability. Meanwhile, the Tri‐WOB provides relatively high bending stiffness when the arm is maintained in a straight, non‐deformed configuration. Although the load‐bearing capacity of the arm remains limited in highly deformed states, the Tri‐WOB design offers a novel strategy to partially decouple deformation capability from load‐bearing performance, and eliminate the continuous energy input, establishing a practical balance between morphing flexibility and structural support.

## 5 Conclusions

This work highlights the potential of bistable origami structures as an effective strategy for achieving variable stiffness in soft robotic systems. Unlike conventional stiffness enhancement approaches that rely on continuous energy input, the Tri‐WOB leverages intrinsic bistability to achieve stiffness modulation without sustained actuation. On the one hand, the large reconfiguration range enables the Tri‐WOB based robotic arm to achieve posture control. In contrast to traditional continuum SRAs that rely on continuous deformation and often suffer from configuration uncertainty, the proposed arm operates within a finite set of stable states, improving repeatability while reducing control complexity. On the other hand, the ability of Tri‐WOB to program spatially non‐uniform stiffness distributions suggests a promising strategy for soft robotic manipulators to maintain structural support while enabling controlled deformation.

Despite these advantages, several limitations remain. Fabrication imperfections can lead to misalignment between opposing side vertices, reducing the effectiveness of vertex engagement and therefore the achievable stiffness. Additionally, while the Tri‐WOB significantly improves load‐bearing capacity in the straight configuration, the load‐bearing capability decreases when large bending deformations disrupt the vertex contact mechanism. These limitations suggest that further improvements in manufacturing precision, structural reinforcement, and geometric optimization could enhance system robustness. In the current prototype, the bistable process of each WOB unit is triggered manually by compressing the central vertex. Although several automated triggering strategies were explored, the added mechanisms either interfered with the large deformation capability of the robotic arm or reduced the structural compactness and reliability of the system. Therefore, automated stiffness switching has not yet been implemented in this work and will be further investigated in future designs.

Moreover, extending the concept to other origami geometries and multistable structures may further broaden the design space. A more general analytical model will be developed to describe these systems, paving the way for a comprehensive design framework that can automatically determine the structural topology and geometric parameters based on specified stiffness requirements and operating conditions.

## 6 Experimental Section

### 6.1 Fabrication of the WOB Unit

**FIGURE 5:**
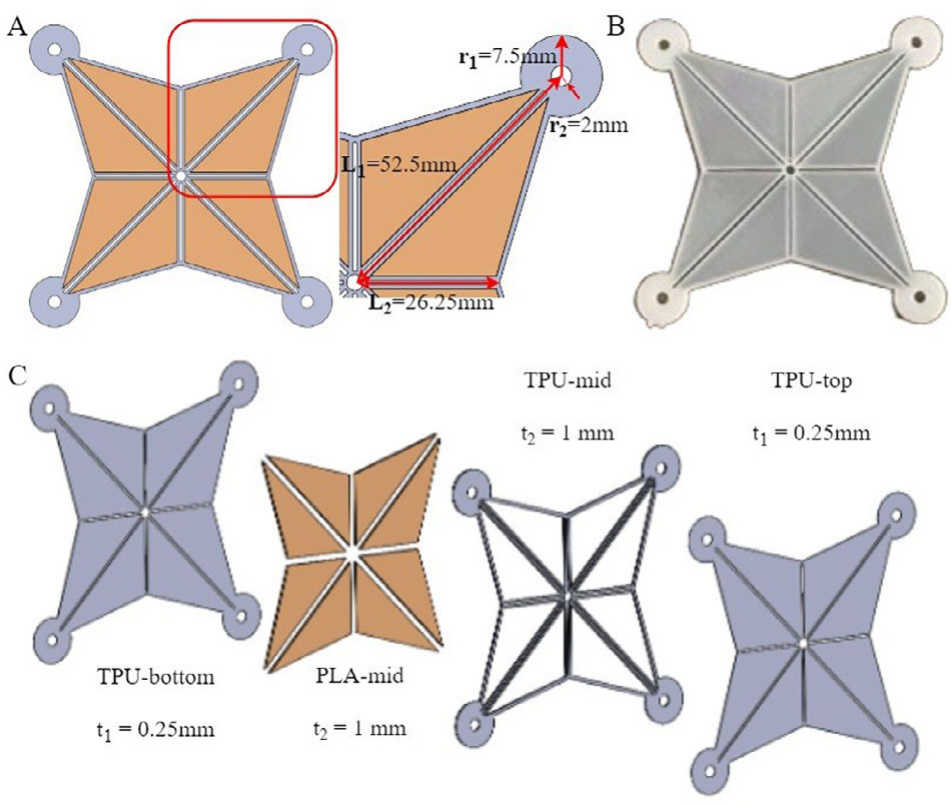
Design and fabrication of the WOB unit. (A) Geometric parameters of the modified WOB unit used in experiments. (B) Fabricated WOB unit. (C) Exploded view of the three‐layer sandwich structure, with two outer TPU layers and a middle layer consisting of PLA panels encapsulated by a TPU frame.

The WOB unit is first modeled using SolidWorks (Dassault Systèmes). To facilitate assembly, a circular feature with a central hole is added at each external foot for fastening to the experimental fixture or the rigid frame of the Tri‐WOB using screws and bolts. The detailed geometric parameters of the WOB unit are provided in Figure 5A. Each WOB unit is designed as a three‐layer sandwich structure composed of two materials, as shown in Figure 5C. The top and bottom layers are made of TPU with a thickness of $t_{1}=0.25$ mm) each. The middle layer has a thickness of $t_{2}=1$ mm and consists of triangular PLA panels surrounded by a TPU frame of the same thickness. This configuration results in the PLA panels being fully encapsulated by TPU material. The overall thickness of the WOB unit is therefore 1.5 mm. Samples are printed using a Qidi I‐Fast dual‐extrusion 3D printer.

### 6.2 Experimental Setup of Bistability Tests

**FIGURE 6:**
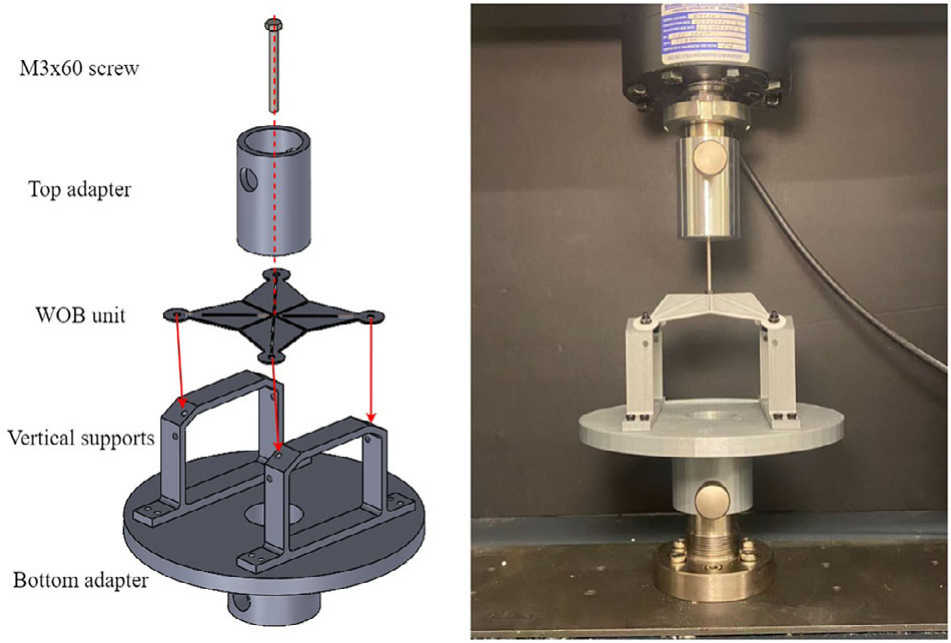
Experimental setup for bistability testing of the WOB unit.

Bistability tests of the WOB unit are performed using a TestResources Universal Testing Machine with custom 3D‐printed PLA fixtures. The central vertex of the WOB unit is connected to the loading head using an M3×60 screw, while the external feet are fixed to vertical supports, which are mounted on a bottom adapter secured to the machine base. The detailed assembly configuration is shown in Figure 6. For each sample, three repeated loading tests are conducted at a compression rate of 5 mm/min to a prescribed displacement of 32 mm. Reaction force and loading‐head displacement are recorded during testing. The testing process is shown in Movie S9.

### 6.3 FEA of the Bistable Process of the WOB Unit

FEA is performed in Abaqus/CAE 2022 to analyze the bistable response of the WOB unit. The WOB unit is modeled in Abaqus as a 3D shell and is partitioned into TPU and PLA regions. For PLA, the Mass Density, Young's Modulus, and Poisson's ratio are set to be 1300 kg/m^3^, 2674 MPa, and 0.3. For TPU, the Mass Density, Young's Modulus, and Poisson's ratio are set to be 1150 kg/m^3^, 35 MPa, and 0.45. All regions are meshed using S4R elements. The whole simulation consists of two steps. The first step (Static, General) allows the WOB unit to fold to gain the initial height. The folded configuration from the first step result is imported as a new model. Then, in the second step (Dynamic, Implicit, Quasi‐static), the folded WOB unit is compressed at the center to trigger the bistable process. To account for fixture compliance, a spring of 10 N/mm is attached to each foot. The reaction force and displacement at the reference point are recorded to obtain the force‐displacement response. The detailed FEA settings can be found in Text S2, and the simulated bistable process is shown in Movie S10.

### 6.4 Experimental Setup of Tri‐WOB Based Robotic Arm

**FIGURE 7:**
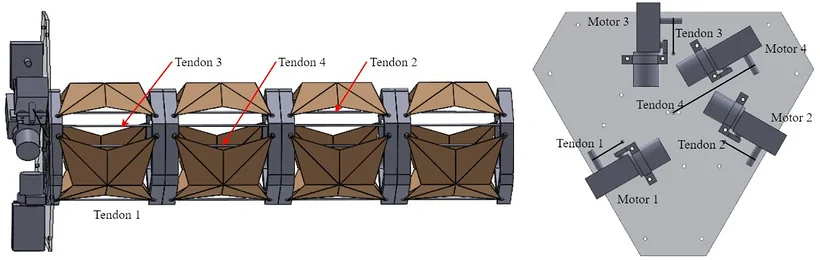
Experimental setup of Tri‐WOB based robotic arm.

The robotic arm consists of four Tri‐WOBs stacked sequentially along the Z‐axis, as shown in Figure 7A. The base is connected to a control module with four motor‐driven actuation units: three motors arrange circumferentially at $120^{\circ }$ intervals for the outer tendons and one motor for the central tendon (Figure 7B). Selective retraction of an outer tendon produces directional bending, whereas retraction of the central tendon induces axial contraction. The control module includes a DC power supply, four 12 V, 30 RPM motors, and four DC motor speed controllers with reversible switches to enable tendon retraction and release. The tendons are wound around the motor shafts so that motor rotation is directly converted into tendon displacement.

## Conflicts of Interest

The authors have no conflicts of interest.

## Supporting information


**Supporting File 1**: advs76898‐sup‐0001‐SuppMat.pdf.

**Supporting File 2**: advs76898‐sup‐0002‐VideoS1‐S10.zip.

## Data Availability

The data that supports the findings of this study are available in the supplementary material of this article
