# Supplementary material for: Tri‐WOB: A Bistability‐Driven Variable Stiffness Origami Structure for Soft Robotic Arms
Source: Adv Sci (Weinh). 2026 Aug 7;13(49):e76898. doi: 10.1002/advs.76898 (PMC13449545; doi:10.1002/advs.76898)
Supplement: Supplementary file 1 [file ADVS-13-e76898-s002.pdf]

Supplementary Material 1 for

Tri-WOB: A Bistability-Driven Variable Stiffness Origami Structure for Soft Robotic Arms

Lingchen Kong

Yaoyao Fiona Zhao*

Department of Mechanical Engineering

McGill University, Montreal, H3A 0G4, Canada

*yaoyao.zhao@mcgill.ca

Supplementary Text

Text S1: Analytical model of the bistable process of the WOB unit

To capture the bistable process of a WOB unit from the 1st stable state to the 2nd stable state, an analytical model is developed based on an inclined beam representation of panel bending. In general, the deformation of a slender beam can be described using the continuous Euler–Bernoulli beam theory, in which the bending moment–curvature relationship is expressed as

$$M(x,y) = EI\kappa = EI\frac{d\phi}{ds}, \tag{S.1}$$

where $M(x,y)$ is the bending moment at a given location along the beam, κ is the local curvature, ϕ, s denote the rotation angle and arc length along the beam. Integrating the above relation, the bending energy of the beam can be written as

$$U = \frac{1}{2}\int EI\kappa^2 ds. \tag{S.2}$$

To simplify the problem, the eight panels of the WOB unit are assumed to deform identically. Each panel is therefore modeled as a multi-segment cantilever beam obtained by discretizing the continuous beam into a series of rigid segments connected by rotational springs (Fig.S.1A). In this discretized representation, the deformed configuration under a prescribed tip position is determined by solving a constrained energy minimization problem.

$$L = \frac{L_{\text{panel}}}{n}, \tag{S.3}$$

where L_{panel} equals to $OA_i = L_1 = 52.5mm$. Let θ_i denote the relative rotation at joint i ($i = 1, \ldots, n$). The absolute orientation of segment i is

$$\phi_i = \sum_{j=1}^{i} \theta_j. \tag{S.4}$$

Under this discretization, the curvature κ in Eq.S.1 can be approximated as

$$\kappa = \frac{\theta_i}{L}. \tag{S.5}$$

Substituting this relation into the Euler–Bernoulli moment–curvature equation yields

$$M(x, y) = EI\kappa = EI\frac{\theta_i}{L}. \tag{S.6}$$

However, directly modeling the WOB panel as a beam with a uniform cross-section may not accurately capture its mechanical response, since the panel width varies along its length. To account for this geometric variation, the local height of the panel is incorporated into the stiffness formulation. Each beam segment is therefore characterized by a local section height h_i, a segment length L, and a constant panel thickness t (Fig.S.1B). Approximating the panel cross-section as rectangular, the second moment of area is given by

$$I_i = \frac{h_i t^3}{12}. \tag{S.7}$$

Accordingly, the effective rotational stiffness of each discretized beam segment can be written as

$$k_i = \frac{EI_i}{L}. \tag{S.8}$$

This formulation allows the bending stiffness to vary along the panel according to the local section height. As a result, segments located in regions with smaller panel heights exhibit lower stiffness and undergo larger rotations under the same bending moment. Such a spatially varying stiffness distribution enables the discretized beam model to capture the natural curvature observed in the deforming WOB panels during the bistable process.

For the WOB unit with different geometric parameters, i.e. different $L1/L2$ ratio, Eq.S.8 requires modification. When the panel thickness remains unchanged, the primary geometric factor influencing the bending stiffness of each segment is the local section height h_i (Fig.S.1C), which makes the rotation stiffness k_i scale linearly with the local section height, i.e. $k_i \propto h_i$. However,

using this linear relationship in the analytical model results in only minor differences between the predicted responses of different geometric configurations, particularly in the maximum force (critical force) associated with the snap-through transition. This behavior does not adequately capture the experimentally observed sensitivity of the bistable response to geometric variations. To better reflect the influence of geometry on the structural stiffness, a nonlinear scaling relationship is introduced. Specifically, the geometry with ratio $L1/L2 = 2 : 1$ is taken as the reference configuration. The corresponding height distribution along the discretized beam is denoted as h_i^{ref}. Then Eq.S.7 can be modified to be

$$I_i = h_i^{\text{ref}} \left(\frac{h_i}{h_i^{\text{ref}}} \right)^{\beta} \frac{t^3}{12}, \tag{S.9}$$

where $\{h_i\}$ denotes the original height of the targeting geometry of the WOB unit and β is an exponent that controls the sensitivity of stiffness to geometric variation. In the present model, $\beta = 1.5$ is adopted. This formulation introduces a nonlinear dependence of the stiffness on the height ratio. When the local height is smaller than that of the reference configuration, the stiffness decreases more rapidly than in the linear case, while larger heights produce a comparatively stronger stiffness increase. As a result, the analytical model becomes more sensitive to geometric variations, allowing it to better capture the differences in bistable behavior observed among WOB units with different $L1/L2$ ratio.

The beam node coordinates are computed recursively. Let (x_0, y_0) denote the base node. Since the base end is fixed, the initial position is

$$x_0 = 0, \qquad y_0 = 0. \tag{S.10}$$

The coordinates of node $i + 1$ are obtained from node i as

$$x_{i+1} = x_i + L\cos(\phi_i), \qquad y_{i+1} = y_i + L\sin(\phi_i), \tag{S.11}$$

where ϕ_i is the absolute orientation of segment i.

However, in practice, the external foot may exhibit a small amount of horizontal compliance due to structural flexibility and assembly tolerance. To account for this effect, the strict constraint $x_0 = 0$ is relaxed by introducing a horizontal spring of stiffness K_h at the base node. In addition, a short portion of the panel near the base is physically clamped to the rigid foot. To represent this boundary condition in the discretized beam model, the rotational stiffness of the first two joints is assigned a very large value in the numerical implementation, which effectively suppresses rotation in this region and prevents vertical displacement near the clamped boundary.

The total potential energy of the system consists of the bending energy stored in the rotational springs and the elastic energy associated with the horizontal compliance of the base fixture:

$$U = U_{\text{bend}} + U_{\text{spring}}. \tag{S.12}$$

The bending energy arises from the rotational springs connecting adjacent beam segments and is expressed as the quadratic energy associated with the deviation of the joint angles from their initial configuration, given by

$$U_{\text{bend}} = \frac{1}{2} \sum_{i=1}^{n} k_i \left(\theta_i - \theta_i^{\text{init}} \right)^2 . \tag{S.13}$$

The horizontal compliance of the experimental fixture is modeled using an equivalent spring attached to the base node. The elastic energy stored in this spring includes both a linear and a weak nonlinear contribution. The linear term represents the small-displacement compliance of the fixture, while the nonlinear term accounts for the gradual increase in resistance observed when the base experiences larger horizontal deformation during the bistable process. The spring energy is therefore written as the sum of a quadratic and a quartic term in the horizontal displacement Δx_0

$$U_{\text{spring}} = \frac{1}{2} K_{h_1} \Delta x_0^2 + \frac{1}{4} K_{h_2} \Delta x_0^4. \tag{S.14}$$

In this formulation, K_{h_1} denotes the linear stiffness of the equivalent fixture spring and characterizes the primary horizontal compliance of the experimental setup. The coefficient K_{h_2} represents the

nonlinear stiffness parameter that introduces a cubic stiffening effect in the restoring force at larger displacements. This additional term allows the model to capture the increased resistance of the fixture under larger deformation while maintaining symmetric energy behavior for positive and negative displacements.

The bistable process is simulated by prescribing the vertical displacement of the tip node while constraining the tip x-coordinate to remain constant, given by

$$x_{\text{tip}} = x_{\text{tip}}^{\text{init}}, \tag{S.15}$$

$$y_{\text{tip}} = y_{\text{tip}}^{\text{init}} + \Delta y, \tag{S.16}$$

where $(x_{\text{tip}}^{\text{init}}, y_{\text{tip}}^{\text{init}})$ denotes the initial position of the tip, determined by the initial configuration of the WOB unit, i.e., the folded state. For each prescribed displacement Δy, the equilibrium configuration is obtained by solving

$$\min_{\{\theta_i\}, x_0} \; U(\{\theta_i\}, x_0) \quad \text{s.t.} \quad \begin{cases} x_{\text{tip}} = x_{\text{tip}}^{\text{init}}, \\ y_{\text{tip}} = y_{\text{tip}}^{\text{init}} + \Delta y. \end{cases} \tag{S.17}$$

The optimization variables are

$$\mathbf{v} = [\theta_1, \theta_2, \ldots, \theta_n, \Delta x_0]^T \,. \tag{S.18}$$

The constrained minimization is solved using Sequential Least Squares Programming (SLSQP) as implemented in SciPy.

The vertical reaction force at the tip is computed from the energy derivative with respect to the imposed displacement:

$$F_y = -\frac{\partial U}{\partial(\Delta y)}. \tag{S.19}$$

Numerically, a central finite difference approximation is used:

$$F_y \approx -\frac{U(\Delta y + \varepsilon) - U(\Delta y - \varepsilon)}{2\varepsilon}, \tag{S.20}$$

where $\varepsilon = 10^{-5}$ in the implementation. For each perturbation, the constrained minimization is re-solved to obtain $U(\Delta y \pm \varepsilon)$.

Text S2: FEA of the bistable process of the WOB unit

FEA is performed in Abaqus/CAE 2022 to study the force-displacement of the bistable process of the WOB unit. The WOB unit is modeled in Abaqus as a 3D shell and partitioned into TPU region (gray) and PLA region (red), as shown in Fig.S.6A. All length variables are consistent with the one discussed in the main body, where $OA_i = L_1 = 52.5mm$ and $OB_i = L_2 = 26.25mm$. The in-plane thickness of the PLA region is $1mm$ and the thickness of the TPU region is $1.5mm$. Since there is no self-interaction, tangential and normal contact are not considered. A gradient mesh is employed to improve accuracy near regions of large stress. The general area is meshed with $0.5mm$ as element size while the circular region around the center is meshed carefully with $0.1mm$ as the element size, as shown in Fig.S.6B. The whole WOB region is meshed by S4R. A linear-elastic material model is used in the simulation. For PLA, the Mass Density, Young's Modulus, and Poisson's ratio are set to be $1300kg/m^3$, $2674MPa$, and 0.3. For TPU, the Mass Density, Young's Modulus, and Poisson's ratio are set to be $1150kg/m^3$, $35MPa$, and 0.45.

A complete simulation of the bistable process can be divided into two stages. In the first stage, the target is to fold the flat WOB unit to the folded state. Therefore, in the first step (Static, General, $1s$), an initial perturbation ($0.1mm$ displacement along the Z axis) is applied to the nodes located in the middle line, with all the feet fixed. Then in the second step (Static, General, $1s$), a $4mm$ displacement is applied to each pair of feet along the Y axis towards the middle line (Fig. S.6C (i)). The folded configuration can be found in Fig. S.6C (ii).

In the second stage, the central region of the WOB unit is compressed to trigger the bistable process. Since the folded configuration obtained in the first stage is defined as the initial state of the bistable process, the stored energy of the system need to be reset to zero. To achieve this, the deformed configuration from the first stage is saved and imported into Abaqus as a new model, as shown in Fig. S.6D. In Step 3 (Dynamic Implicit, Quasi-static), the total step time is set to $30s$. The

initial size of increment, the minimum size of increment, and the maximum number of increments are 0.1, $1e^{-10}$, and 10000, respectively.

A displacement of -32mm (displacement-controlled compression) is applied along the Z-axis to a reference point (RP_1) coupled with all central nodes. To account for potential compliance in the experimental fixture, one end of a spring with stiffness $10N/mm$ is attached to each foot of the WOB unit along the X-axis, while the other end is fixed to the ground. Accordingly, the U_1 degree of freedom of the feet is released, while all other degrees of freedom are constrained. The reaction force and displacement at RP_1 are recorded to obtain the force–displacement curve. The simulated bistable process of a WOB unit can be found in Fig. S.6E (i)-(v). Stress concentration is observed at the panel corners near the fixed feet and at the corners adjacent to the central region.

Text S3: Cyclic testing of the bistability of the WOB unit

The experimental setup for the cyclic test is the same as that used for the bistability test, while the loading conditions are modified. The loading rate is set to 100 mm/min to accelerate the cyclic testing, and the total number of cycles is set to 1000. Each complete cycle consists of a compression step and a tension step, corresponding to the downward and upward movements of the loading head, respectively. A 3 s pause is introduced between the two steps to allow energy dissipation and release. The force–displacement curves are recorded for both the compression and tension steps, and the results are plotted in Fig.S.5.

Text S4: Kinematic modeling of the Tri-WOB using a 3-RSR mechanism

The bending behavior of a single Tri-WOB is modeled as a 3-RSR mechanism and simulated in Adams. The arrangement of the three link pairs is defined to match the distribution of the tendons, such that each link pair is aligned with one tendon, as shown in Fig. S.8A. In this model, the upper and lower bars are connected to the top and bottom rigid frames, respectively, through revolute joints, while a spherical joint is used between each corresponding pair of upper and lower bars. To simulate bending, a rotational input is applied to the front link pair while the top frame is fixed.

During the simulation, the position of the bottom frame is recorded throughout the motion. Since the focus here is on the end posture of the bent Tri-WOB, only the coordinates at the last time step are used for subsequent analysis.

Since only one tendon is actuated, the Tri-WOB remained symmetric with respect to Plane H (i.e., the XOZ Plane, as shown in Fig. S.8B). Therefore, when projecting the motion of the top and bottom frames to Plane H, only the X and Z coordinates are considered. The intermediate and final projected posture of the bent Tri-WOB in Plane H is shown in Fig. S.8C (i) and (ii), respectively. Fig. S.8D is then generated in Python using the final coordinates of the top and bottom frames extracted from the Adams simulation, and therefore represents the projected end posture of the bent Tri-WOB in State 000. In contrast, when the Tri-WOB is in State 111, corresponding to the high-stiffness state, the structure does not deform under tendon contraction. In this case, the bottom frame remains directly below the top frame for $H = 84mm$ without rotation.

When four Tri-WOB units are stacked to form the robotic arm, the arm has a total of 16 (= 2^4) possible configurations. For each Tri-WOB configuration, the position of the bottom frame is known relative to the top frame. Starting from the base Tri-WOB, the top frame is fixed and taken as the initial reference. Once the configuration of the first Tri-WOB is specified, the position of its bottom frame can be determined. Since the top frame of the second Tri-WOB is connected to the bottom frame of the first unit, the end position of the second bottom frame can then be calculated based on the selected configuration of the second unit. The same procedure is repeated for the third and fourth Tri-WOB units. In this way, the final projected posture of the entire robotic arm in Plane H can be obtained.

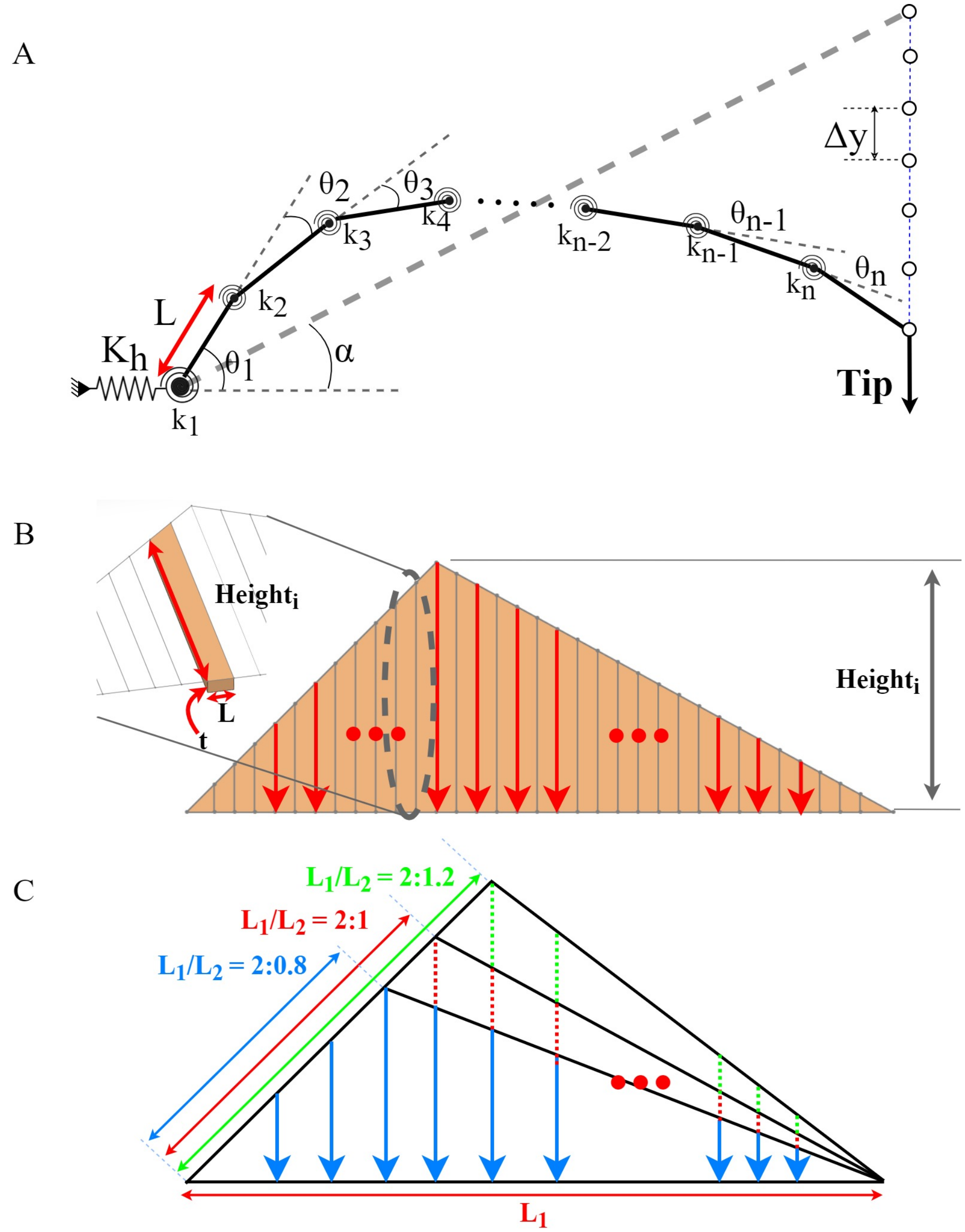

Figure S.1: **Analytical model of the WOB unit.** (**A**) Multi-segment cantilever beam representing a panel of the WOB unit. (**B**) Panel height variation along the beam. (**C**) Geometry variation of ratio L_1/L_2

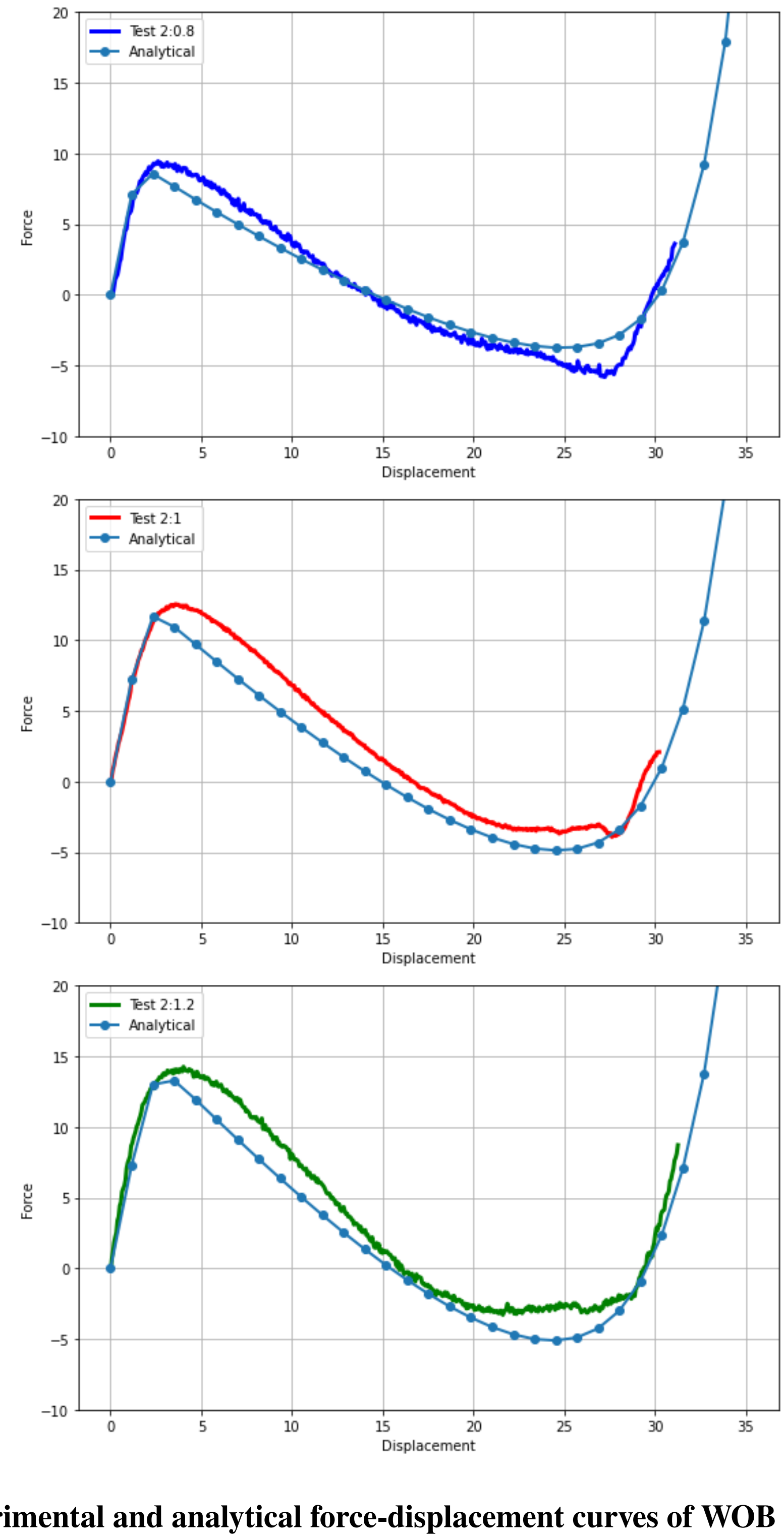

Figure S.2: **Experimental and analytical force-displacement curves of WOB units with varying geometric ratio L_1/L_2 when thickness $t_2 = 0.8mm$.**

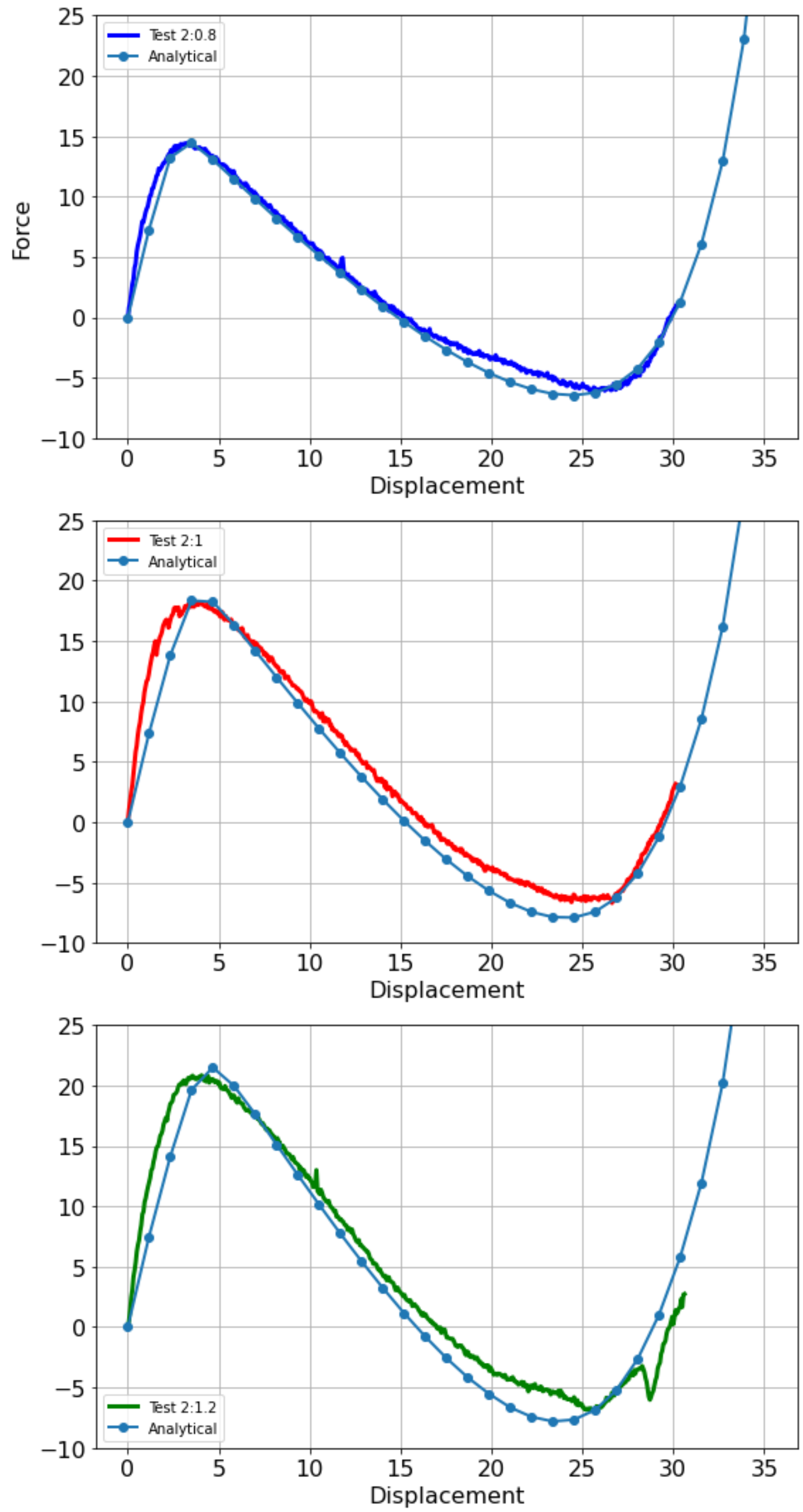

Figure S.3: **Experimental and analytical force-displacement curves of WOB units with varying geometric ratio L_1/L_2 when thickness $t_2 = 1mm$.**

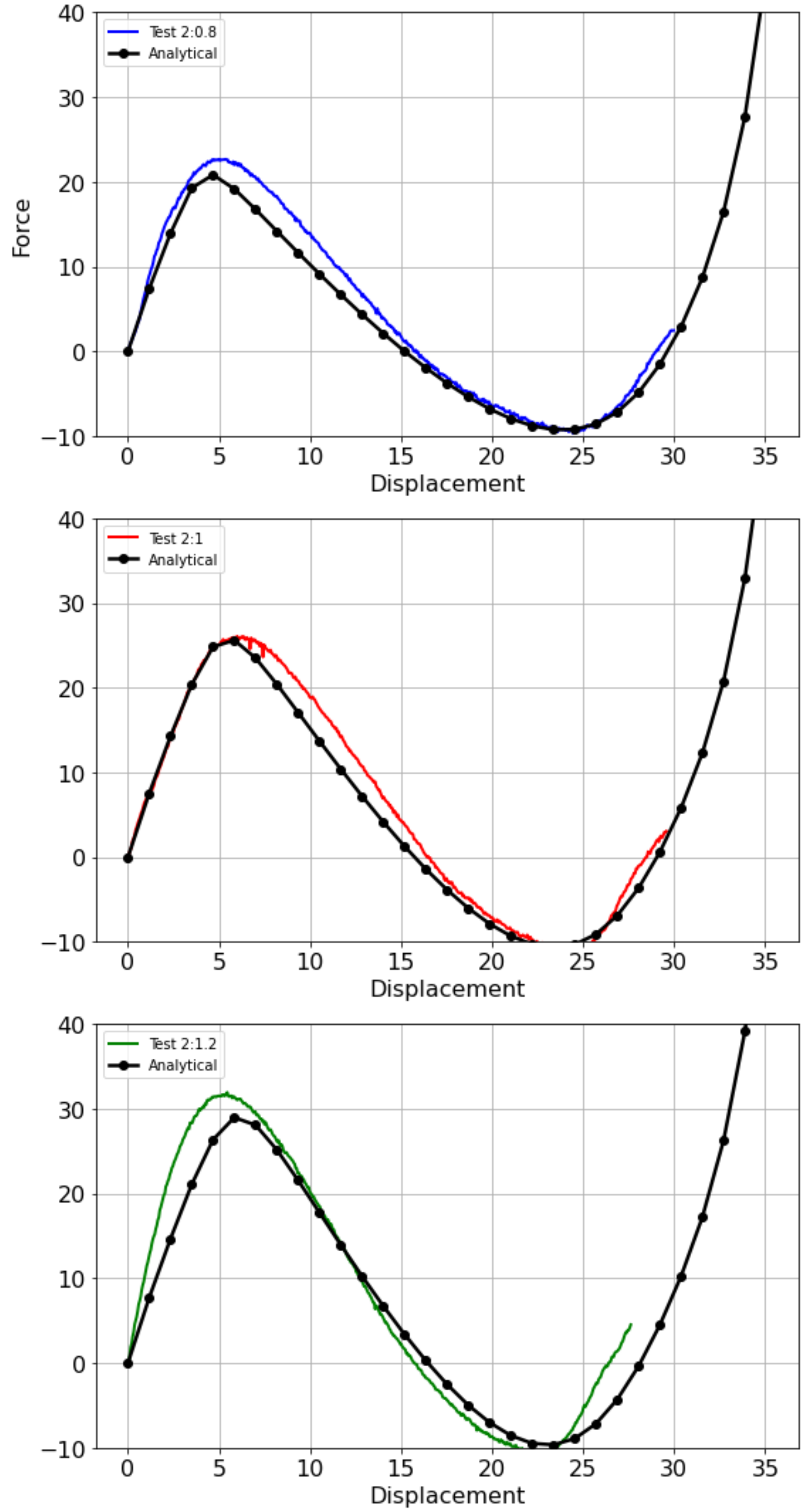

Figure S.4: **Experimental and analytical force-displacement curves of WOB units with varying geometric ratio L_1/L_2 when thickness $t_2 = 1.2mm$.**

A)

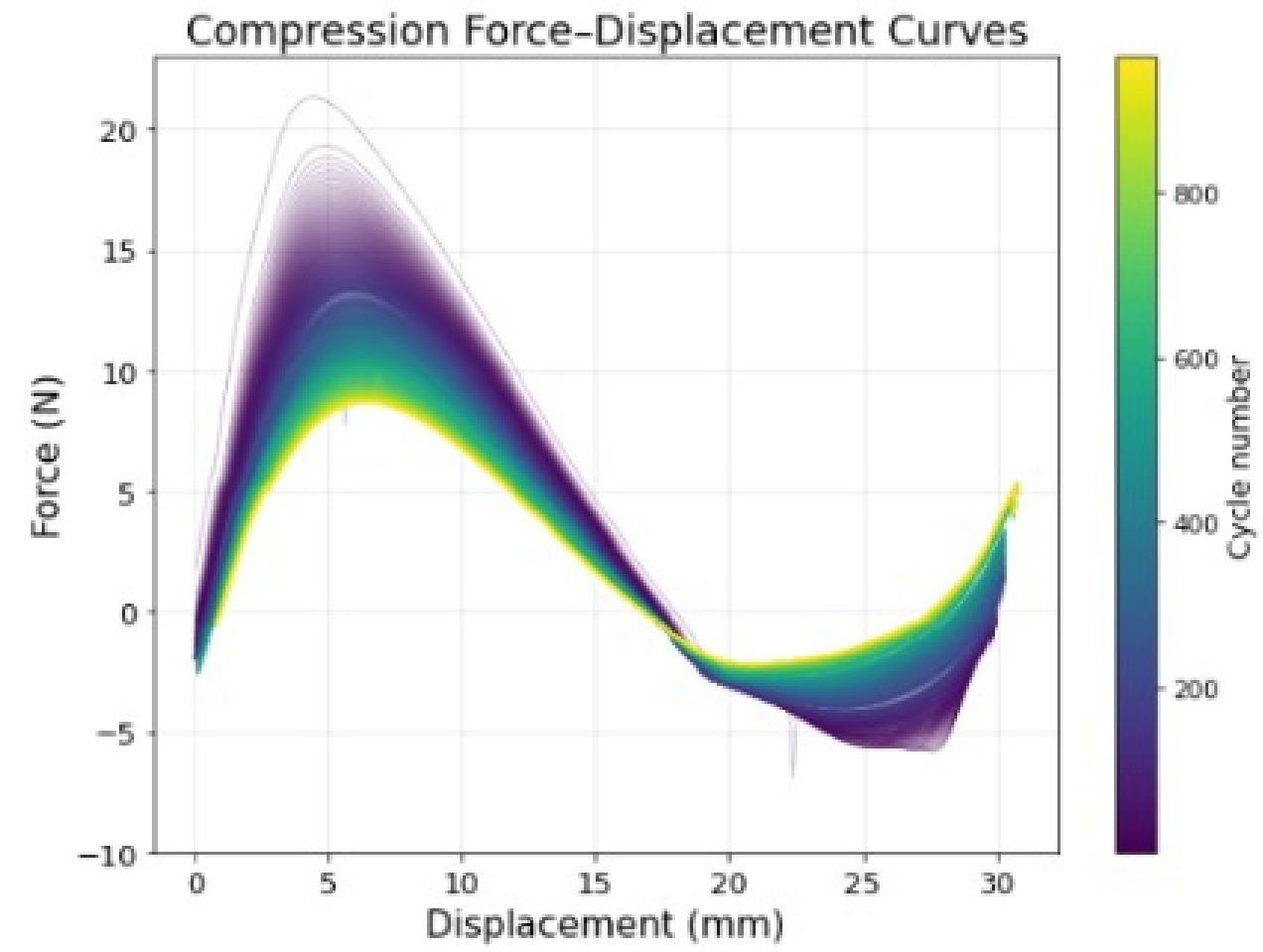

B)

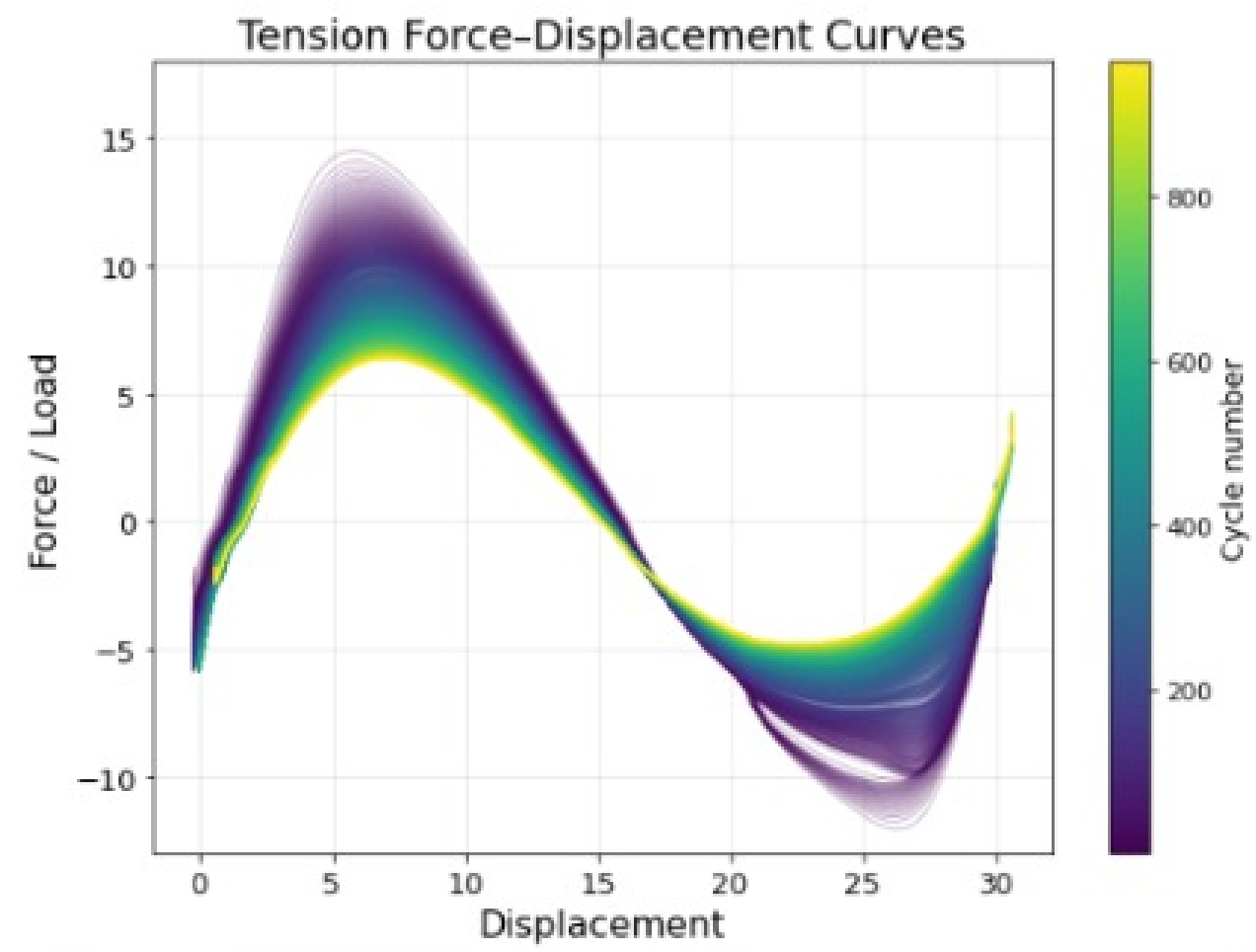

C)

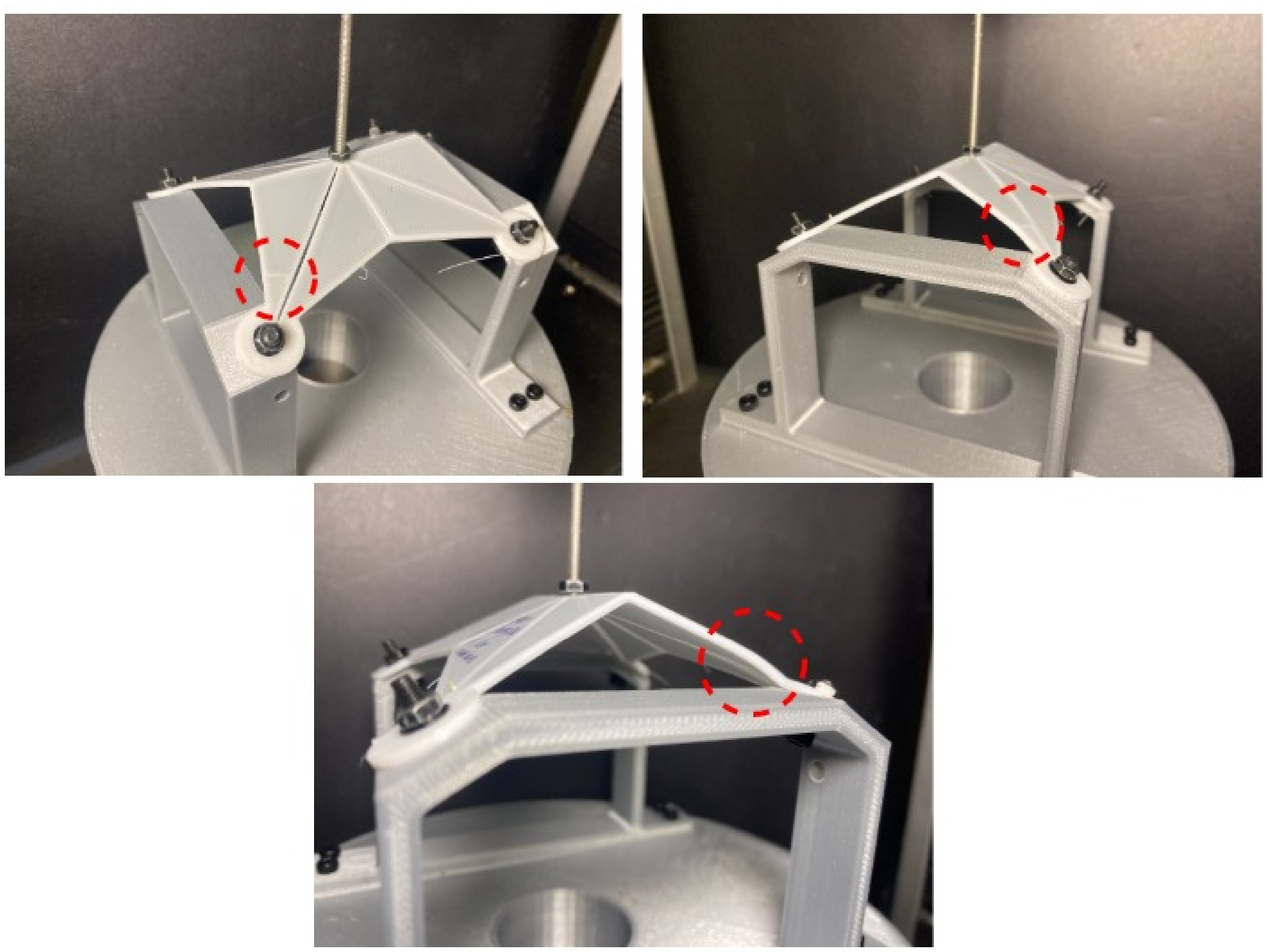

Figure S.5: **Force-displacement curves of 1000-cycle bistability test.** (**A**) Compression. (**B**) Tension. (**C**) Plastic deformation around the foot section of the WOB unit after 1000 cycles.

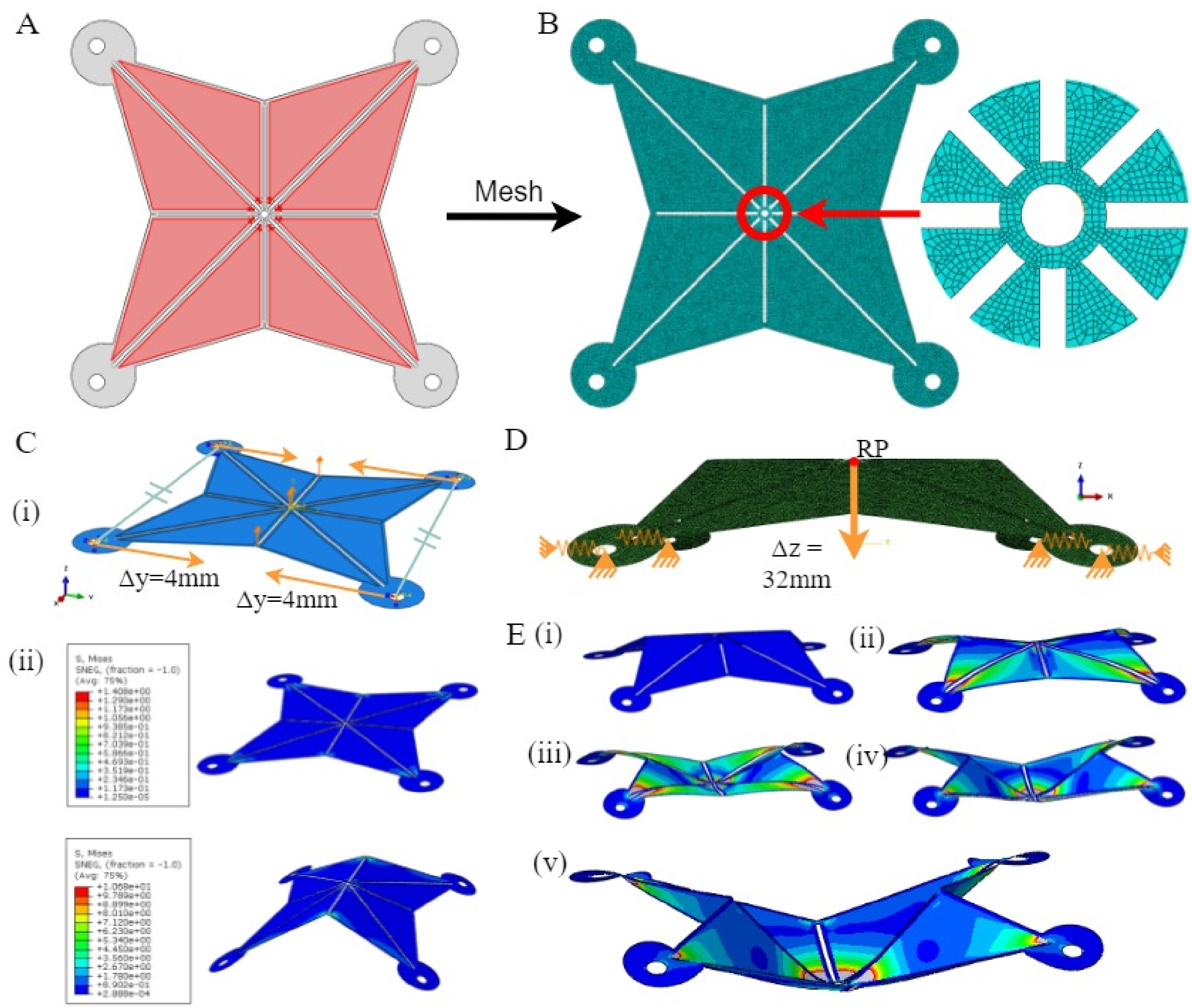

Figure S.6: **Finite element analysis of the bistable process of the WOB unit.** (**A**) Abaqus model of the WOB unit with TPU and PLA regions. (**B**) Gradient mesh used in the simulation, with local refinement near the central region. (**C**) First simulation stage for folding the flat WOB unit: (i) applied boundary conditions; (ii) resulting folded configuration. (**D**) Second simulation stage, where the folded configuration is imported as a new model and subjected to displacement-controlled compression at the center. (**E**) Simulated bistable process of the WOB unit.

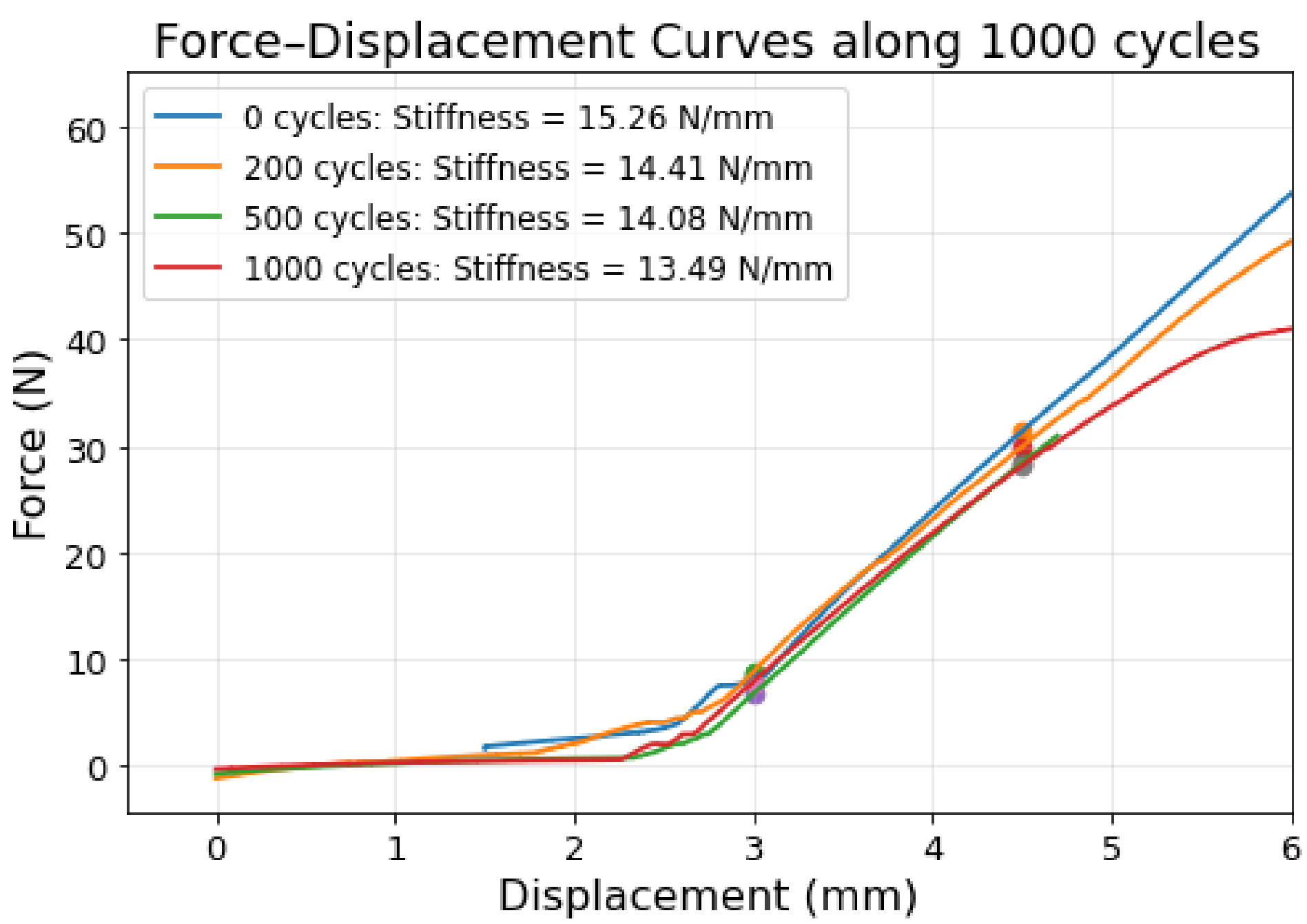

Figure S.7: **Force–displacement curves of the Tri-WOB assembled from WOB units after 0, 200, 500, and 1000 loading–unloading cycles.**

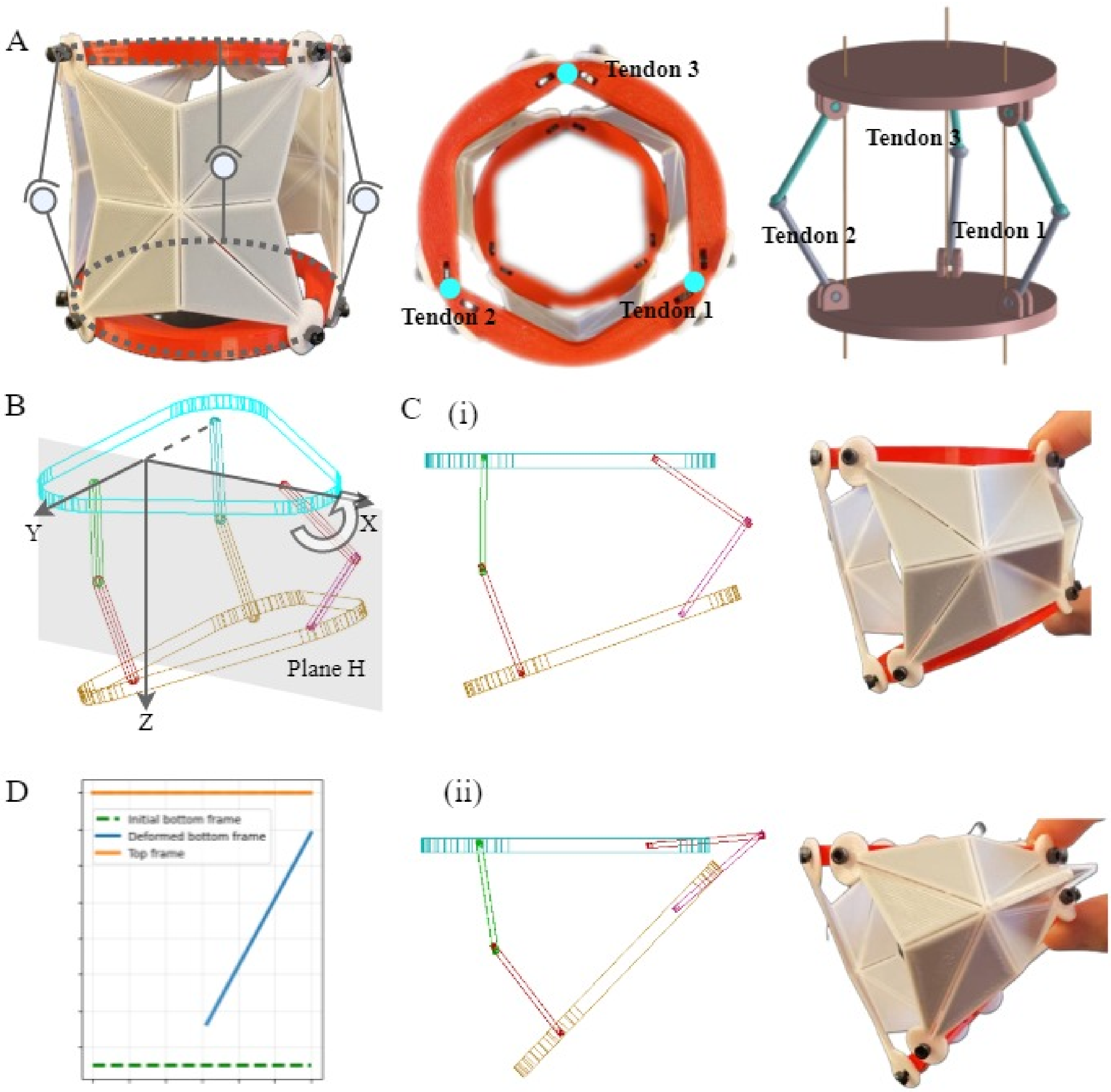

Figure S.8: **Kinematic modeling of a single Tri-WOB using a 3-RSR mechanism.** (**A**) Schematic of the 3-RSR mechanism, showing the three link pairs aligned with the tendon positions. (**B**) Adams model of the 3-RSR mechanism. (**C**) Projected views of the bent Tri-WOB in Plane H: (i) intermediate posture; (ii) end posture. (**D**) Python-generated projected view reconstructed from the Adams-exported final coordinates, corresponding to State 000.

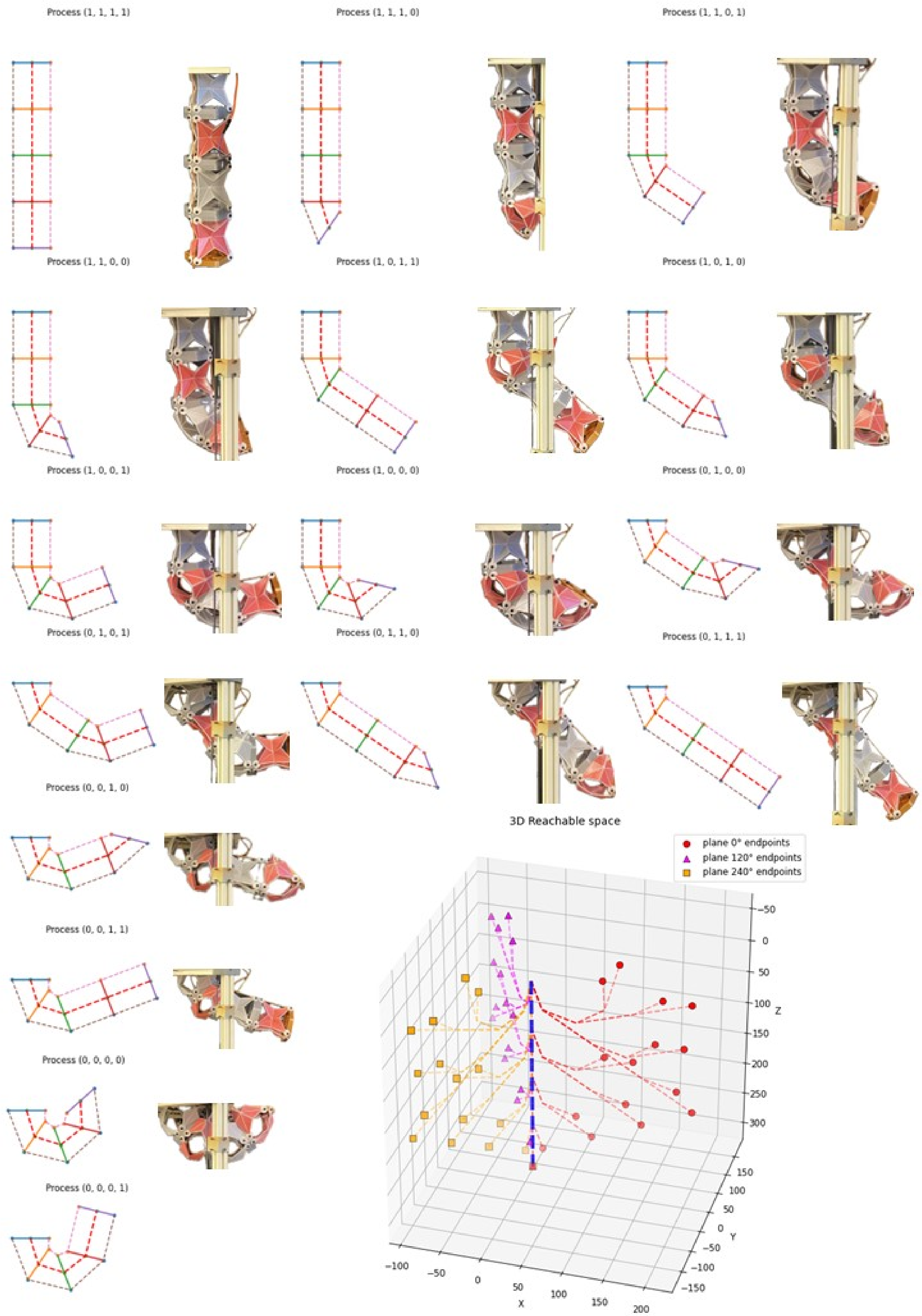

Figure S.9: **End postures of the 16 configurations of the Tri-WOB-based robotic arm.** For the last configuration, the distal module is predicted to rise far above the base level. The presence of the top plate prevents it from reaching this position. Thus, no experimental photograph is shown. The lower right figure shows the 3D reachable space of the robotic arm.

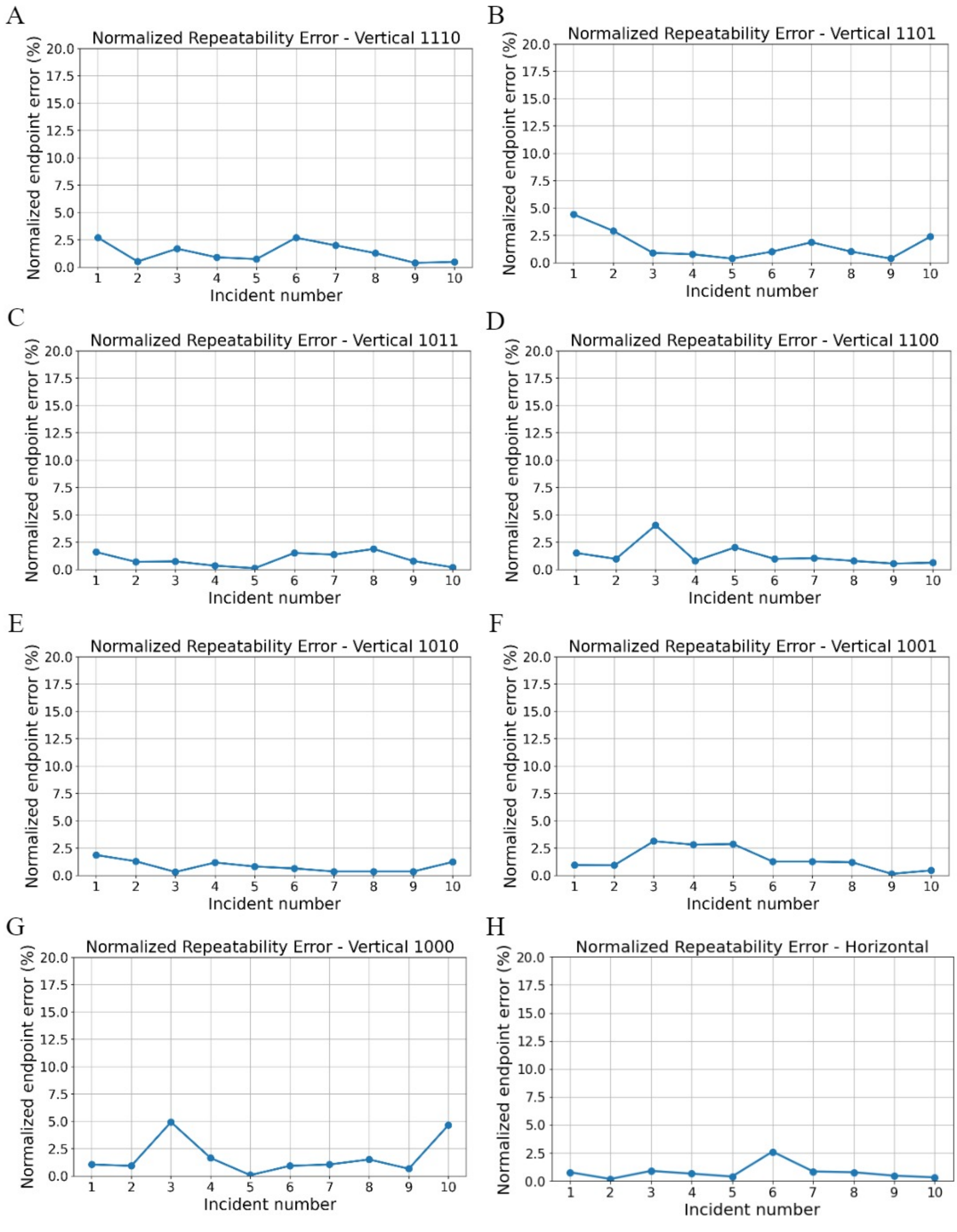

Figure S.10: **Repeatability analysis**. (**A**)-(**G**) Vertically oriented robotic arm under seven representative configurations. (**H**) Horizontally oriented robotic arm.

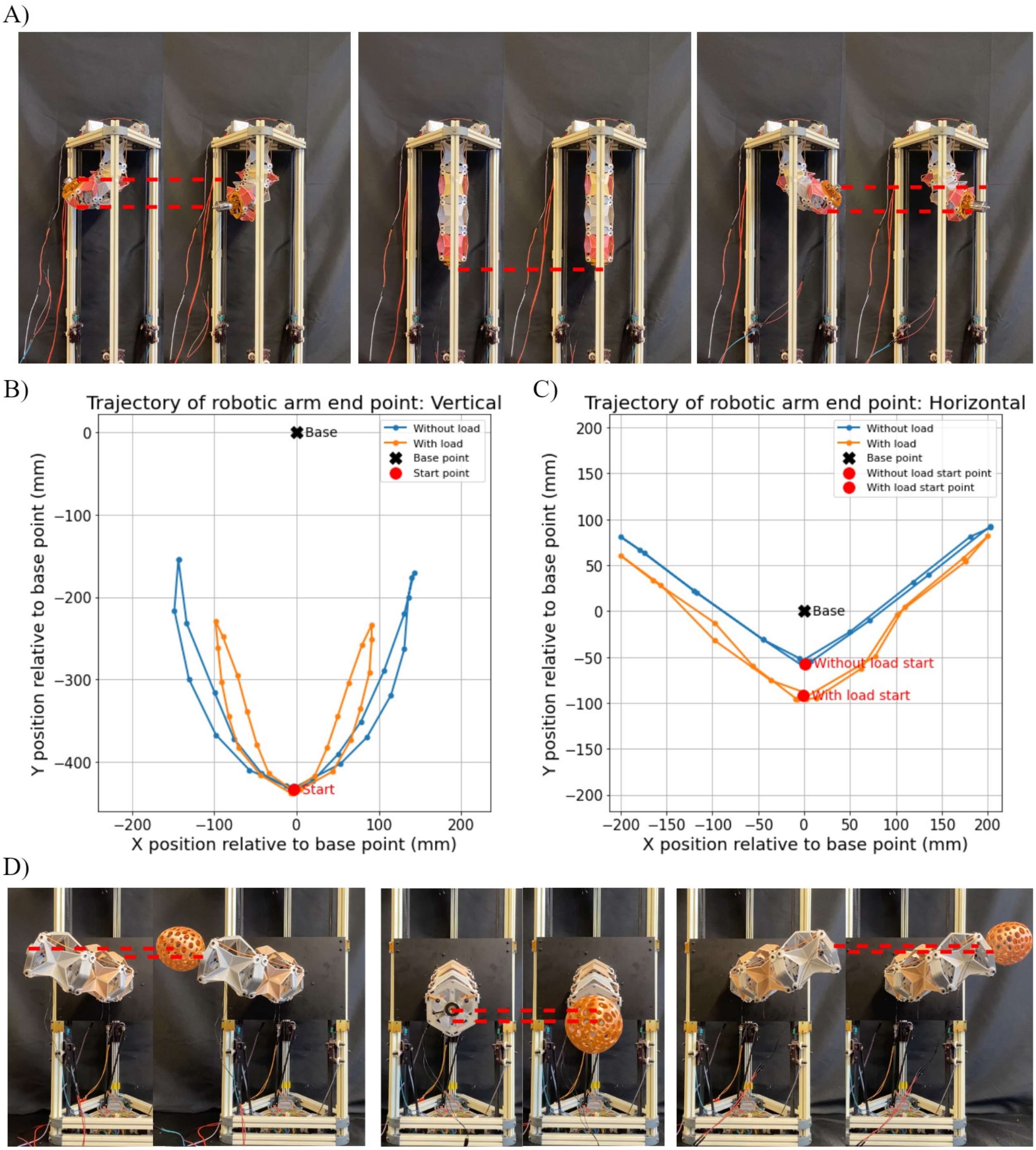

Figure S.11: **Trajectory-holding capability under load**. (**A**) Position shifting of the vertically oriented robotic arm for bent and initial states with/without load. (**B**) Trajectory of the vertically oriented robotic arm for bent and initial states with/without load. (**C**) Trajectory of the horizontally oriented robotic arm for bent and initial states with/without load. (**D**) Position shifting of the horizontally oriented robotic arm for bent and initial states with/without load.

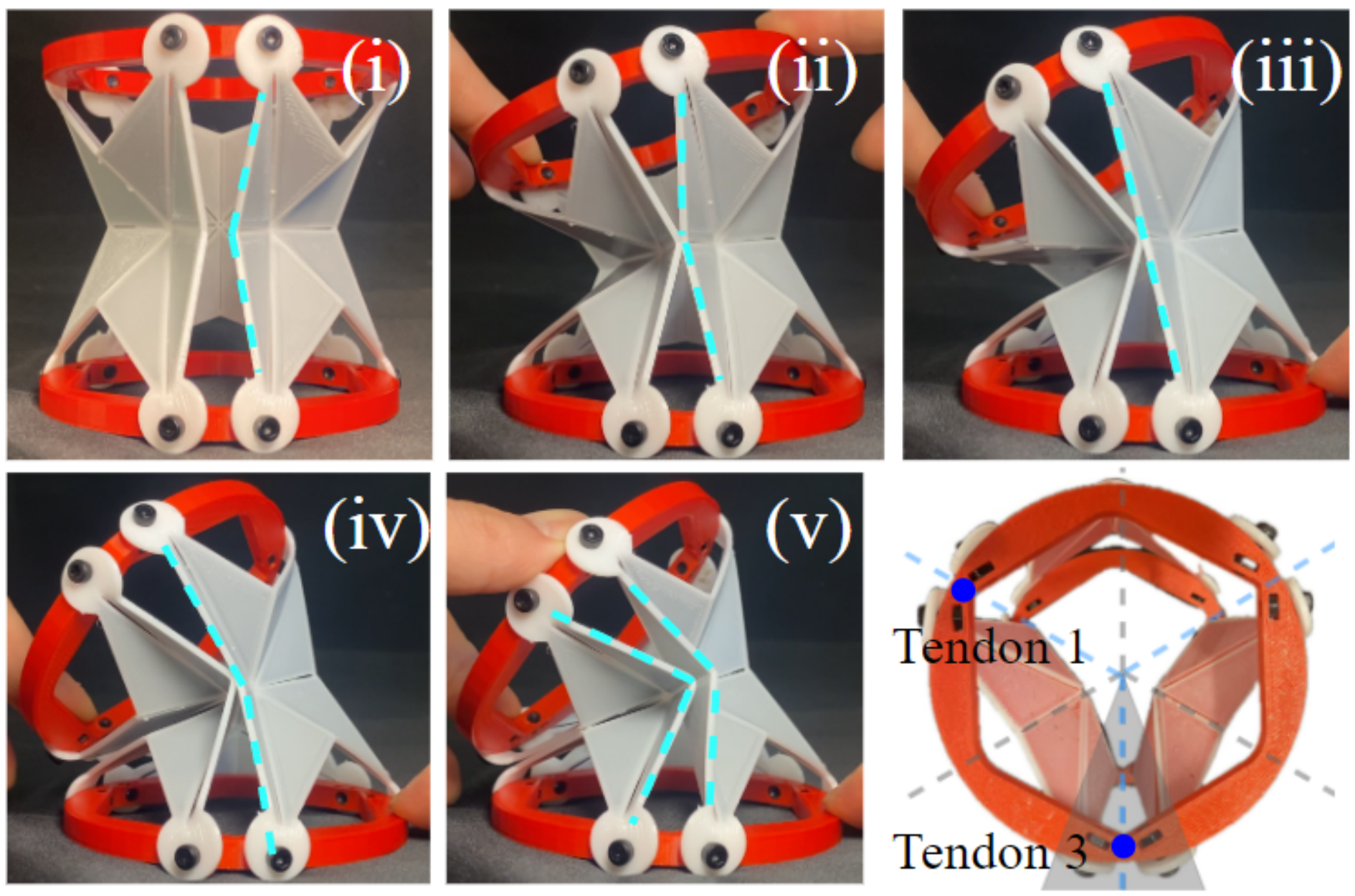

Figure S.12: **Stiffness compromise under directional bending in State 101**: (i) initial state; (ii) bending along Tendon 1 enlarging the "<" shape; (iii) critical "|" alignment; (iv) inverts to the shape ">"; (v) aligned "> >" shape.

Table S1: Comparison of stiffness-enhancing strategies in soft robotic arms and manipulators.

Strategy	Stiffness enhancing range/ratio	Load capacity	Response	Hold energy	Deformation range	Repeatability	Ref.
Chain jamming	0.55 N - 5.09 N 10× lateral	1 kg	–	Yes	90% axial elongation; 90° bending	–	[S1]
Granular jamming	21–71 $N\,m^{-1}$; 3.4×	1.5 N	0.5 s	Yes	–	–	[S2]
Tendon–pneumatic coupling	–	0.05–1.00 kg	–	Yes	30.78 mm contraction; 59.96° bending; 33.67° twisting	10-cycle SD: 0.15 mm, 0.22°, 0.13°	[S3]
Pneumatic chamber stiffening	>7× lateral	0.5–1 kg	–	Yes	3× axial extension; 3-DoF bending	0.19–1.27 mm trajectory error	[S4]
Pneumatic facet buckling	> 300×	356 g lateral	–	Yes	3.1× compression; 120° bending	–	[S5]
Pneumatic origami gripper	–	1 kg	–	Yes	3-DoF contraction/ extension/bending	Max. tracking error: 8 mm	[S6]
Pneumatic + antagonistic actuation	–	2.5–4.4 N; 1.8×	–	Yes	3-DoF motion; 200% extension	1.42 mm mean positioning error	[S7]
Pneumatic + antagonistic actuation	>278%	600 g	–	Yes	Large S-shaped bending workspace	2.54% error-to-length ratio	[S8]
Antagonistic actuation	23.9–542.5 $N\,m^{-1}$	0.07–2.17 kg lateral	1.06 Hz	Yes	Large S-shaped bending workspace	–	[S9]
SMA friction locking	1.41–1.50×	–	18–115/60 s heat/cool	Yes	–	3.5–5.4% error	[S10]
SMA friction locking	0.0129–0.0370 $N\,mm^{-1}$; 2.87×	3 N	16/22–29 s heat/cool	Yes	Large S-shaped bending workspace	<2.23% endpoint error	[S11]
LMPA phase change	0.56–2.47×	50 g lateral; 200 g axial	2/2.5 min heat/cool	No	90.2° bending	2.17% locked recovery	[S4]
LMPA phase change	45×	1 kg	85/12 s heat/cool	No	Minimum bending radius ≈50 mm	–	[S12]
LMPA phase change	–	>20 g at 90° locked bend	13–15.7/5–10 s heat/cool	No	Near-90° bending in eight directions	1.37° angle SD over 10 repeats	[S13]
**Bistable Origami**	**234.8× axial; >100× lateral**	**50 N axial; 5 N lateral**	–	**No**	**180° arc bending; 4× axial contraction**	**<5% max. error over 10 cycles**	**This work**

Notes: LMPA = low-melting-point alloy; SMA = shape-memory alloy. "Hold energy" indicates whether continuous input is required to maintain the stiff state. SD = standard deviation.

Movie S1

Bistable process of the Tri-WOB and demonstration of stiffness increase

Movie S2

Compression test for the Tri-WOB in high stiffness state

Movie S3

Local variable stiffness of the Tri-WOB: mechanical response of the Tri-WOB in State 100 and State 101

Movie S4

Free morphing capability of the Tri-WOB: Axial contraction and J-type bending

Movie S5

The grasping and load transportation capabilities of the vertically oriented robotic arm

Movie S6

Bending stiffness change of the Tri-WOB based robotic arm

Movie S7

The Tri-WOB based robotic arm loads an object while bending

Movie S8

The grasping and load transportation capabilities of the horizontally oriented robotic arm

Movie S9

Compression test for the bistable process of the WOB unit

Movie S10

Numerical simulation of the bitable process of the WOB unit

References

[S1] Z. Xie, M. Mohanakrishnan, P. Wang, J. Liu, W. Xin, Z. Tang, L. Wen, C. Laschi, *IEEE Robotics and Automation Letters* **2023**, *8*, 6 3597.

[S2] L. Al Abeach, S. Nefti-Meziani, T. Theodoridis, S. Davis, *Journal of Bionic Engineering* **2018**, *15*, 2 236.

[S3] J. Wang, H. Yang, J. Zhang, H. Liu, Y. Zhong, Y. Hu, Y. Liu, C. Xia, J. Wu, *Advanced Intelligent Systems* **2025**, *7*, 8 2401008.

[S4] Z. Zhang, G. Chen, Y. Xun, Y. Long, J. Wang, H. Wang, J. Angeles, *IEEE Transactions on Robotics* **2023**, *39*, 6 4768.

[S5] M. Park, W. Kim, S.-Y. Yu, J. Cho, W. Kang, J. Byun, U. Jeong, K.-J. Cho, *IEEE Robotics and Automation Letters* **2022**, *8*, 2 864.

[S6] Z. Zhang, W. Fan, Y. Long, J. Dai, J. Luo, S. Tang, Q. Lu, X. Wang, H. Wang, G. Chen, *Cyborg and Bionic Systems* **2024**, *5* 0103.

[S7] Z. Zhang, S. Tang, W. Fan, Y. Xun, H. Wang, G. Chen, *Mechanism and Machine Theory* **2022**, *169* 104607.

[S8] E. Harsono, J. Yang, S. Bhattacharya, H. Yu, *Mechanism and Machine Theory* **2022**, *177* 105067.

[S9] J. Zhang, C. Yang, H. Yang, P. Ma, C. He, T. Zhang, X. Wang, K. Liu, X. Jia, H. Peng, *Nature Communications* **2026**.

[S10] H. Jeon, Q. N. Le, S. Jeong, S. Jang, H. Jung, H. Chang, H. J. Pandya, Y. Kim, *IEEE Robotics and Automation Letters* **2022**, *7*, 3 6582.

[S11] C. Yang, S. Geng, I. Walker, D. T. Branson, J. Liu, J. S. Dai, R. Kang, *The International Journal of Robotics Research* **2020**, *39*, 14 1620.

[S12] Z. Xing, F. Wang, Y. Ji, D. McCoul, X. Wang, J. Zhao, *IEEE robotics and automation letters* **2020**, *6*, 2 755.

[S13] C. Hu, J. Liu, J. Chen, S. Dai, J. Wang, B. Zhang, H. Liao, *IEEE Robotics and Automation Letters* **2023**, *8*, 9 5704.
